# Sustained‐Release Ulcer‐Protective Microarray Patch With Dihydrocapsaicin for Diabetic Wound Regeneration

**DOI:** 10.1002/EXP.20250129

**Published:** 2026-05-28

**Authors:** Yuxu Wu, Kan Jiang, Xiao Wen, Chihao Jin, Kai Zhang, Jiakai Zhang, Jiale Jin, Jiaqian Wang, Huiyu Hu, Zonghan Xu, Xianzhu Zhang, Yihe Hu

**Affiliations:** ^1^ Department of Orthopedic Surgery The First Affiliated Hospital Zhejiang University School of Medicine Hangzhou China; ^2^ Department of Radiation Oncology The First Affiliated Hospital Zhejiang University School of Medicine Hangzhou China; ^3^ Department of Burns and Plastic Surgery The Affiliated Suzhou Hospital of Nanjing Medical University, Suzhou Municipal Hospital Gusu School of Nanjing Medical University Suzhou China; ^4^ Department of Orthopedic Zhongshan Hospital Fudan University Shanghai China; ^5^ Center for Systems Biology Massachusetts General Hospital Boston Massachusetts USA; ^6^ Department of Orthopedic The Affiliated Suzhou Hospital of Nanjing Medical University, Suzhou Municipal Hospital Gusu School of Nanjing Medical University Suzhou China

**Keywords:** angiogenesis, diabetic foot ulcers, diabetic wound, dihydrocapsaicin, metal‐organic frameworks, microneedle, reactive oxygen species

## Abstract

Diabetic foot ulcers (DFUs) present a persistent clinical challenge, posing a significant and ongoing threat to patient health. Current treatment strategies are failing to concurrently address the excessive oxidative stress and impaired angiogenesis during whole‐course management of diabetic wounds. To overcome these limitations, this study developed an innovative microneedle (MN)‐based drug delivery system incorporating cerium‐based metal‒organic frameworks (MOFs) loaded with dihydrocapsaicin (DHC). Fabricated from a Gelatin Methacryloyl (GelMA) hydrogel, the resulting MN‐MOF@DHC system exhibited a sustained release profile of DHC that nearly covered the whole‐course management of diabetic wounds. Our results revealed that the MN‐MOF@DHC system effectively reduced the levels of reactive oxygen species (ROS), promoted the transformation of macrophage phenotypes from the pro‐inflammatory M1 subtype to the anti‐inflammatory M2 subtype, and significantly enhanced endothelial cell angiogenesis. These combined actions markedly accelerated wound closure. Collectively, the advanced MN platform offers a highly promising strategy for the treatment of DFUs.

## Graphical abstract

Schematic illustration of the effect of microneedles incorporating cerium‐based MOF loading dihydrocapsaicin (MN‐MOF@DHC) to scavenge reactive oxygen species (ROS) generation, beanti‐inflammatory, inhibit the apoptosis of endothelial cells (ECs), promote angiogenesis, and accelerate diabetic wound healing.

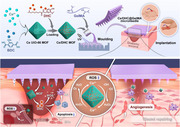

## 1 Introduction

Diabetes afflicts nearly 529 million people globally, with its prevalence increasing annually [1, 2]. Diabetic foot ulcers (DFUs) represent one of the most prevalent and severe complications of diabetes, carrying significant risks of persistent pain, delayed wound recovery, limb amputations, and potentially premature mortality [3, 4]. Current clinical management of DFU relies on antibiotics for infection control, traditional dressings for moisture regulation, and growth factor therapies to stimulate repair [4, 5]. However, antibiotics face rising resistance rates, while dressings lack bioactive functionality. Growth factors exhibit short half‐lives and fail to steadily work in the whole‐course treatment of DFU [6]. The lack of effective therapeutic approaches for DFUs represents a significant clinical challenge.

Wound healing is a meticulously coordinated biological process including three overlapping phases: inflammation, proliferation, and remodeling that depends on the complex interaction of diverse biomolecules, cellular elements, signaling pathways, and matrix production [7, 8, 9, 10]. The delayed healing observed in diabetic wounds can be attributed to multiple factors, including high levels of oxidative stress and impaired angiogenesis during the whole‐course management of diabetic wound [11, 12, 13, 14, 15]. The pathological microenvironment of diabetic wounds is characterized by the abnormal accumulation of reactive oxygen species (ROS). This oxidative imbalance directly drives endothelial apoptosis and microvascular regression, both critical barriers to healing [13, 16, 17, 18]. The excessive ROS in diabetic wounds further intensifies local inflammatory responses and prevents macrophages from switching from the proinflammatory M1 status to the reparative M2 status, thus delaying the regenerative process [19, 20]. Moreover, persistent hyperglycemia exacerbates this condition by increasing the generation of advanced glycation end products (AGEs), which negatively impact the formation of vascular endothelial growth factor (VEGF), thereby disrupting angiogenesis and wound closure [21]. Collectively, these factors underscore the need for a therapeutic platform that can synergistically mitigate oxidative stress, alleviate inflammation, and promote angiogenesis.

Dihydrocapsaicin (DHC), the primary active component of capsaicin found in plants belonging to the genus Capsicum, has a range of therapeutic effects, including antioxidative, anti‐inflammatory, antiapoptotic, and proangiogenic effects, as demonstrated in rat cerebral ischemia–reperfusion (I/R) models [22, 23, 24]. The mechanism of its protective effects on endothelial cells is multifaceted: it elevates the synthesis of nitric oxide (NO) along with endothelial nitric oxide synthase (eNOS), markedly diminishes the TNF‐α‐triggered expression of adhesion molecules such as VCAM‐1 and ICAM‐1, curbs IL‐6 generation, and impedes the activation of NFκB [25]. Additionally, DHC functions as an AKT signaling activator, increasing VEGF expression and promoting local angiogenesis through the activation of the PI3K/AKT signaling cascade [26]. Lin et al. reported that DHC effectively inhibits oxidative stress and NLRP3 inflammasome activation by blocking the cGAS–STING signaling pathway in perforator flaps [27]. This dual action not only curtails detrimental processes but also stimulates angiogenesis, underscoring its potential therapeutic efficacy for treating diabetic wounds. DHC monotherapy, despite its well‐documented anti‐inflammatory, antioxidant, and antimicrobial properties, faces challenges related to poor solubility, low bioavailability, and rapid clearance when administered conventionally [28]. These limitations significantly restrict its therapeutic efficacy in chronic wound settings where prolonged and targeted action is required. Therefore, developing a novel drug delivery system to extend its beneficial impacts on diabetic wound healing is crucial.

Owing to the low fluctuation of drug concentrations in the blood, convenient administration, and high patient adherence, transdermal drug administration has become one of the primary drug administration routes in contemporary medicine [29, 30, 31]. One important requirement for successful transdermal drug delivery is increasing skin permeability to improve drug bioavailability. Diverse strategies have been suggested for this purpose, including the use of nanoparticles [32], pressurization [33], electric simulation [34], and microneedles [35, 36, 37]. Among these, microneedles stand out because of their ability to penetrate the tissue deeply without causing significant injury or damage due to their sharp tips and microscale sizes [38, 39, 40]. In addition, microneedles offer flexibility in material choice, enabling effective transport of hydrophilic, lipophilic, and other types of drugs [37, 38, 41, 42]. Despite these advantages, existing MN platforms, while effective in bypassing the skin barrier and enabling transdermal drug delivery, often suffer from limited drug‐loading capacity, insufficient mechanical strength for penetrating diabetic skin, and the inability to provide sustained and long‐term release of therapeutic agents [43]. Moreover, many current MNs lack multifunctionality, such as simultaneous antioxidant and antimicrobial activity, which is critical for addressing the complex microenvironment of diabetic wounds [44]. Therefore, there is a need for advanced microneedle systems that can regulate local microenvironments and provide sustained drug release for the whole‐course management of diabetic wounds.

Metal‐organic frameworks (MOFs), composed of organic connectors and metallic nodes, are distinguished by their molecular‐level catalytic sites, elevated porosity, and superior loading capabilities [45, 46]. The adaptable architecture of MOFs endows them with diverse shapes and chemical properties, whereas the fragile ligand linkages ensure their biodegradability. Moreover, MOFs synergistically regulate ROS levels and increase ATP production [20, 47, 48, 49]. These unique features make MOFs highly promising materials for drug delivery applications. Among all kinds of MOFs, a previously screened cerium‐based MOFs (Ce‐MOFs) with optimized ligands exhibiting superior ROS‐scavenging and drug‐loading capabilities was selected to achieve controlled and sustained drug release. Furthermore, integrating Ce‐MOFs into Gelatin Methacryloyl (GelMA) microneedles mimics the natural extracellular matrix ​​and​​ promotes cellular proliferation within the microneedle structure, thereby making this system particularly suitable for treating diabetic wounds [50].

In this study, we designed a GelMA microneedle system integrated with Ce‐MOFs (MN‐MOF) for loading DHC (Scheme 1). This novel MN‐MOF@DHC system exhibited stable local DHC delivery over an extended period and demonstrated significant potential in clearing ROS. Our results showed that this composite effectively attenuated oxidative stress damage and inflammation, inhibited apoptosis, and promoted vascular regeneration, ultimately accelerating the healing of diabetic wounds.

## 2 Materials and Methods

### 2.1 Synthesis of MOF@DHC

The synthesis of Ce‐UiO‐66‐CH_3_ was carried out using a modified procedure based on a reported method [51]. Firstly, in a 10 mL Pyrex glass reaction tube, 0.23 mmol of 2‐methyl‐1,4‐benzenedicarboxylic acid (BDC‐CH_3_) (Aladdin, China) was mixed with 0.5333 mmol of cerium (IV) ammonium nitrate (Aladdin, China) dissolved in 1.2 mL N,N‐dimethylformamide (DMF) (Aladdin, China). The sealed glass reactor was then heated to 100°C while being continuously stirred for a duration of 30 min. After allowing it to cool down to room temperature, the remaining product was harvested through centrifugation and thoroughly rinsed 3 times using acetone. 10 mg of Ce‐UiO‐66‐CH_3_ was introduced into 10 mL of DHC solution (ID0590, Solarbio, China), and the mixture was softly agitated overnight. The final Ce‐MOF and MOF@DHC were subjected to centrifugation at 4,000 rpm for 10 min at a temperature of 4°C, employing a refrigerated centrifuge to maintain their structural stability and avert any untimely release of the drug.

### 2.2 Preparation of MN‐MOF@DHC

A 10% solution of GelMA (with 60% substitution, supplied by EFL, China) was prepared, containing deionized water and a photo‐initiator. MOF or MOF@DHC was directly introduced into the GelMA solution and stirred overnight. The hydrogel solution was introduced into a microneedle template (EFL, China), and the cavities were filled with the solution through vacuum processing. Following the elimination of excess hydrogel solution, the loaded microneedle underwent photopolymerization via UV exposure (365 nm, 6.9 mW cm^−2^). In the end, the template was removed, and then we got the MN‐MOF or MN‐MOF@DHC.

### 2.3 Material Characterization

The morphological characteristics of MOF and MN‐MOF, as previously mentioned, were examined using transmission electron microscopy (TEM), which was operated at an accelerating voltage of 100 kV, along with scanning electron microscopy (SEM) operated at an accelerating voltage of 10 kV. The crystal structure of the Ce‐MOF and MOF@DHC was subjected to phase analysis by X‐ray diffraction (XRD) analyzer under the following test conditions: copper target (λ = 0.15418 nm), tube voltage of 40 kV, tube current of 25 mA, scanning speed of 0.5°/min, and scanning range of 5°‐130°. Fourier transform infrared spectroscopy (FTIR) data were also acquired (Thermo Fisher, USA).

### 2.4 Catalase Assay

An H_2_O_2_ solution with a concentration of 100 × 10^−6^ M H_2_O_2_ was mixed with 10 µg/mL^−1^ Ce‐UiO‐66‐CH_3_ for 5 min. Then the decomposition of H_2_O_2_ by catalase was swiftly terminated by adding ammonium molybdate. The residual H_2_O_2_ then reacted with the ammonium molybdate, resulting in the formation of a pale‐yellow complex. The absorbance of the resultant complex was determined at a wavelength of 405 nm using a UV–vis spectrophotometer.

### 2.5 Superoxide Dismutase (SOD) Assay

The 10 µg/mL^−1^ Ce‐UiO‐66‐CH_3_ nanozymes, 0.02 U/mL^−1^ XO and 0.15 × 10^−3^ M X were mixed together for 5 min. Subsequently, WST‐1 was introduced into the composite solution. Oxygen radicals (O_2_^−^) present in the solution can interact with WST‐1, leading to the formation of formazan, which can be obtained at a 450 nm utilizing a UV‐vis spectrophotometer.

### 2.6 Hydroxyl Radical Scavenging Assay

A concentration of 100 × 10^−6^ M H_2_O_2_ undergoes a Fenton reaction to generate hydroxyl radicals. After being co‐incubated with the 10 µg/mL^−1^ Ce‐UiO‐66‐CH_3_ for 5 min, the Griess reagent was subsequently induced to the composite solution. The amount of hydroxyl radicals generated exhibits a direct proportionality to the absorbance value determined at a wavelength of 550 nm.

### 2.7 Dihydrocapsaicin (DHC) Release Detection

The microneedles (MN@DHC and MN‐MOF@DHC) were submerged in 50 mL of PBS solution. At the predetermined time point, the PBS solution of 200 µL was withdrawn from the tube and replaced with an equal volume of fresh PBS. To determine the release rate of DHC, ferric chloride (3 mM) was added to the collected PBS sample. The resulting solution was then measured using a multi‐scan spectrum at 485 nm from Biotek (Manchester, USA).

### 2.8 Mechanical Strength Tests

Utilizing a universal testing machine, the pressure‐displacement curve along with the stress‐time curves were accurately plotted. The microneedles/hydrogels were positioned on the stationary bottom platform of the machine. Subsequently, the mobile upper platform, equipped with a sensor, slowly moved towards the microneedles with a speed of 1 mm/min^−1^ or towards the hydrogels with a speed of 1 cm/min^−1^. Throughout the entire course of this process, the real‐time changes in force were precisely and systematically recorded.

### 2.9 Swelling Rate Test

The microneedle was positioned inside a sealed container containing 20 mL of PBS maintained at 37°C. Once the specified time period had elapsed, the hydrogel underwent weighing procedures 3 times. The swelling ratio was then computed using the formula (W_t_—W_0_) / W_0_ × 100%, where W_0_​ signifies the initial weight of the hydrogel, while W_t_ represents the weight of the hydrogel subsequent to the predefined swelling period.

### 2.10 Cell Culture

RAW 264.7 cells were purchased from the ATCC cell bank and cultured in RPMI‐1640 medium (JYK‐SJ‐001, Inner Mongolia Jinyuankang Biotechnology Co., Ltd., China) supplemented with 10% fetal bovine serum (FBS; BaiDi Biotechnology Co., Ltd. (BDBIO), China) and 1% penicillin‐streptomycin solution (Wuhan Procell Biotechnology Co., Ltd., China). The culture medium was refreshed every 2–3 days.

Human umbilical vein endothelial cells (HUVECs) were obtained from the Chinese Academy of Sciences and maintained in high‐glucose DMEM (BioChannel Biological Technology Co., Ltd. China) supplemented with 10% FBS (KX‐A1233, Inner Mongolia Wanrui Biotechnology Co.,Ltd., China) and 1% penicillin‐streptomycin solution (Wuhan Procell Biotechnology Co., Ltd., China). In order to mimic the pathological oxidative stress environment typically found in diabetic wound tissue, HUVECs were treated with a concentration of 100 µM tert‐butyl hydroperoxide (TBHP) [52].

### 2.11 Cytotoxicity of Microneedles

Cell viability was assessed with the Cell Counting Kit‐8 (CCK‐8; BLT, China) in HUVECs. Each group (Control, DHC, MN‐MOF, and MN‐MOF@DHC) was seeded with HUVECs in a 24‐well plate and cultivated at 37°C in a humidified environment with 5% CO_2_. The viability of HUVECs was assessed after being treated with different concentrations of drug for 24 h, following the guidelines provided by the manufacturer of the CCK‐8. Additionally, the effects of MN‐MOF, DHC, and MN‐MOF@DHC on cell vitality were examined using Live‐Dead Cell Staining Kit I (APExBIO, Houston, USA) on days 1, 3, and 5. The cells were subjected to the specified treatments and examined under a fluorescence microscope (Olympus, Japan) to assess their viability and morphology.

### 2.12 ROS Levels in Cell and Mitochondria

The ROS‐scavenging test was employed primarily in accordance with the method described in a previous study [53]. At a density of 2.5 × 10^5^ HUVECs per well, we were inoculated into a 24‐well culture plate. The ROS levels in cell and mitochondria were assessed using the DCFH‐DA assay kit (Servicebio, China) and the MitoSOX Red Mitochondrial Superoxide Indicator kit (Beyotime, China) following the manufacturer's guidelines. The cells were rinsed with PBS and subsequently incubated in serum‐free medium containing 10 µM DCFH‐DA at 37°C for 20 min. The cells were examined and visualized through a fluorescence microscope (Olympus, Japan), and then the fluorescence intensity was measured utilizing ImageJ software (Version 1.53, NIH, USA). The staining procedure for MitoSOX is analogous to the one employed for DCFH‐DA.

### 2.13 Measurement of Mitochondrial Membrane Potential

The transmembrane potential of mitochondria was evaluated utilizing the JC‐1 Mitochondrial Membrane Potential Assay Kit (AC12L072, Life‐iLab, China). After washing the HUVECs twice with PBS, they were exposed to the JC‐1 dye and maintained at 37°C for a period of 30 min. The HUVECs were examined under a fluorescence microscope. The ratio of red‐to‐green fluorescence intensity was measured with ImageJ (Version 1.53, NIH, USA).

### 2.14 TUNEL Staining

After treatment, HUVECs were subjected to fixation with 4% paraformaldehyde (PFA) for a duration of 20 min and subsequently stained using the One Step TUNEL Apoptosis Assay Kit (Beyotime, China) according to the manufacturer's instructions. Cell nuclei were stained with 4′,6‐diamidino‐2‐phenylindole (DAPI) (Beyotime, China). Positively stained cells were then detected using a confocal microscope (SP8, Leica, Germany).

### 2.15 Western Blot

Total cellular proteins were extracted utilizing RIPA lysis buffer (HAKATA, China), which was supplemented with protease inhibitor cocktail (HAKATA, China). The protein concentrations were precisely quantified employing the BCA protein assay kit (Solarbio, China). Equal quantities of protein (10 µg) were then resolved via 4%‐20% sodium dodecyl sulfate‐polyacrylamide gel electrophoresis (SDS‐PAGE) gels (GenScript, China), followed by transfer onto polyvinylidene fluoride (PVDF) membranes. The membranes were subjected to blocking in a 5% skimmed milk solution for 1 h at room temperature. After that, they were washed by enhanced TBST buffer (Swiss Affinibody LifeScience AG, China) and then incubated with primary antibodies overnight at 4°C. The primary antibodies used were anti‐Cleaved Caspase‐3 (ET1602‐47, HUABIO, China), anti‐Bax (ET1603‐34, HUABIO, China), anti‐Bcl‐2 (ET1702‐53, HUABIO, China), and anti‐β‐actin (HA722023, HUABIO, China). On the following day, the membranes were incubated with the appropriate secondary antibodies at room temperature for a duration of 1 h. The protein bands were then made visible using an enhanced chemiluminescence (ECL) kit (Shandong Sparkjade Biotechnology Co., Ltd., China) and analyzed using ImageJ software (Version 1.53, NIH, USA).

### 2.16 Transwell Assay

A quantity of 2 × 10^4^ HUVECs per well was seeded into the upper chamber of Transwell inserts (NEST Biotechnology, China) with 200 µL of serum‐free culture medium. The lower compartment was added with 500 µL of complete culture medium. Following incubation for a duration of 12 h at 37°C in an incubator containing 5% CO_2_. A cotton swab was used to get rid of the non‐migrated HUVEC cells in the upper chamber. Then, we gently rinsed the HUVECs that had migrated to the underside of the membrane with PBS. After that, we fixed them with 4% PFA for 20 min and stained with crystal violet solution for 30 min.

### 2.17 Tube Formation Assay

The experimental procedures primarily followed the method previously described in report [54] and are briefly outlined here. HUVECs (2 × 10^4^ cells per well in a 96‐wells plate) were utilized for the tube formation assay. The HUVECs were cultivated on a substrate coated with Matrigel (Cat#NM‐G005‐HC, Novoprotein, Shanghai, China), embedded within a hydrogel matrix. Following a 12‐hour incubation period, images of the formed tubes were obtained using a microscope, and subsequently analyzed quantitatively using ImageJ (Version 1.53, NIH, USA).

### 2.18 Immunofluorescence Staining

The experimental cells were seeded in 24‐well plates, and following exposure to various stimuli. The culture medium was eliminated through aspiration, and thereafter, the cells underwent a triple‐wash procedure with PBS. Subsequently, the cells were subjected to fixation using 4% paraformaldehyde (PFA) for a period of 20 min at room temperature. Then they underwent permeabilization treatment with 0.1% Triton X‐100 (abs9149, absin, China) for 30 min and blocking with a 5% BSA (Coolaber, China) solution for 60 min at room temperature. After that, cells were subjected to an overnight incubation at 4°C with primary antibodies: CD31 (11265‐1‐AP, Proteintech, China) and VEGF (19003‐1‐AP, Proteintech, China) for HUVECs, and anti‐iNOS (ab178945, Abcam, USA), anti‐CD206 (ab64693, Abcam, USA), and anti‐F4/80 (ab6640, Abcam, USA) for RAW 264.7 cells. On the next day, the cells were subjected to an incubation process with fluorescently‐conjugated secondary antibodies for a period of 2 h at room temperature. The cytoskeleton of the cells was labeled with phalloidin (Yeasen, China), and the cell nucleus was stained with DAPI (Beyotime, China) for 15 min. Lastly, the cells were subjected to observation under a fluorescence microscope (SP8, Leica, Germany), and the intensity of fluorescence was quantified using ImageJ software (Version 1.53, NIH, USA).

### 2.19 ELISA

To examine the influence of DHC, MN‐MOF, and MN‐MOF@DHC on macrophages, the concentrations of Interleukin‐4 (IL‐4), Interleukin‐10 (IL‐10), and Interleukin‐13 (IL‐13) in the cell culture supernatants were quantified utilizing ELISA kits (Shanghai hengyuan biological technology co., LTD., China). The determined concentrations were subsequently derived based on standard curves.

### 2.20 Quantitative Real Time Polymerase Chain Reaction (qRT‐PCR)

In brief, total RNAs were isolated utilizing TRIzol reagent (Invitrogen, USA). Subsequently, the RNAs underwent reverse transcription into cDNA using the SteadyPure Universal RNA Extraction Kit (ACCURATE BIOTECHNOLOGY (HUNAN) CO., LTD., Changsha, China) strictly adhering to the provided operational guidelines. qRT‐PCR was then carried out employing UltraStart SYBR Green qPCR Master Mix (EXONGEN, Chengdu, China) in accordance with the manufacturer's protocols, with GAPDH serving as the internal control. The primer sequences were devised based on PubMed resources and synthesized by GENCEFE Biotech (Table S1).

### 2.21 Evaluation of Wound Healing in Vivo

The Ethics Committee at the author's institute granted ethical approval for the animal experiments. Male Sprague Dawley (SD) rats, with an age of 6 weeks and a body weight range of approximately 200–250 g, were selected as the experimental model to conduct an in vivo assessment of wound healing processes. The rats were first fed a high‐fat diet (HFD) for a duration of 4 weeks. The HFD consisted of 60% kcal from fat (primarily lard and soybean oil), 20% from carbohydrates, and 20% from protein, matching the standardized formulation for inducing insulin resistance in rodent models [55]. Subsequently, they received a one‐time intraperitoneal injection of streptozotocin (STZ; SJ‐MA0060, Sparkjade, China), which was freshly prepared at a dose of 50 mg/kg in a 0.1 M citrate buffer (pH 4.5), to induce diabetes. A week later, rats with fasting blood glucose levels exceeding 16.7 mM were identified as having type II diabetes. These diabetic rats were then switched to a normal diet and maintained on it for an additional 8 weeks. Subsequently, the type II diabetic rats were randomly assigned into 4 groups (n = 3): Control, MN@DHC, MN‐MOF, and MN‐MOF@DHC. Rats were anesthetized with 3% sodium pentobarbital (60 mg/kg), after which a full‐thickness skin defect of 15 mm diameter was created on the back and 6 mm diameter on the foot. Photographic images were taken to visually document the wound area on days 0, 4, 8, 12, and 16 after surgery, and ImageJ software was used for quantitative analysis of the wound tissue. To assess in vivo wound healing, both hematoxylin‐eosin (H&E) staining and Masson's trichrome staining methods were applied.

### 2.22 Immunohistochemistry and Immunofluorescence in Vivo

To examine angiogenesis, inflammation, and apoptosis in the process of wound healing, tissue samples were extracted and then fixed with 4% PFA for 7 days. Tissue sections from the wounds underwent immunohistochemical staining to detect CD31, as well as immunofluorescence staining for both CD31 and α‐SMA. Vessel density was measured with ImageJ software. In addition, immunofluorescence staining for iNOS, CD206, Bax, VEGF, and Ang‐1 (bs‐0800R, Bioss, China) was performed to measure the levels of inflammation as well as apoptosis in the diabetic wounds. The quantitative analysis of all immunofluorescence stainings was performed using ImageJ software (Version 1.53, NIH, USA).

### 2.23 Dihydroethidium (DHE) Staining

The ROS levels within diabetic wound tissue were evaluated with DHE staining. To begin, the diabetic wound tissues were sectioned into 20 µm thick frozen slices. These tissue samples underwent incubation with a DHE solution for 30 min at a temperature of 37°C. After three washes with PBS, the cell nuclei were subsequently stained using DAPI for a period of 15 min at ambient room temperature. Finally, the sections were imaged using a confocal laser microscope (SP8, Leica, Germany). ImageJ software was used to measure the fluorescence intensity.

### 2.24 Statistical Analysis

The quantitative results were presented as mean values accompanied by standard deviations (SD). Statistical evaluations were carried out employing one‐way ANOVA, with the threshold for significance established at *p* < 0.05. To guarantee the robustness and replicability of the results, every experiment was performed at least three times.

## 3 Results and Discussion

### 3.1 Synthesis and Characterization of MN‐MOF@DHC

**FIGURE 1:**
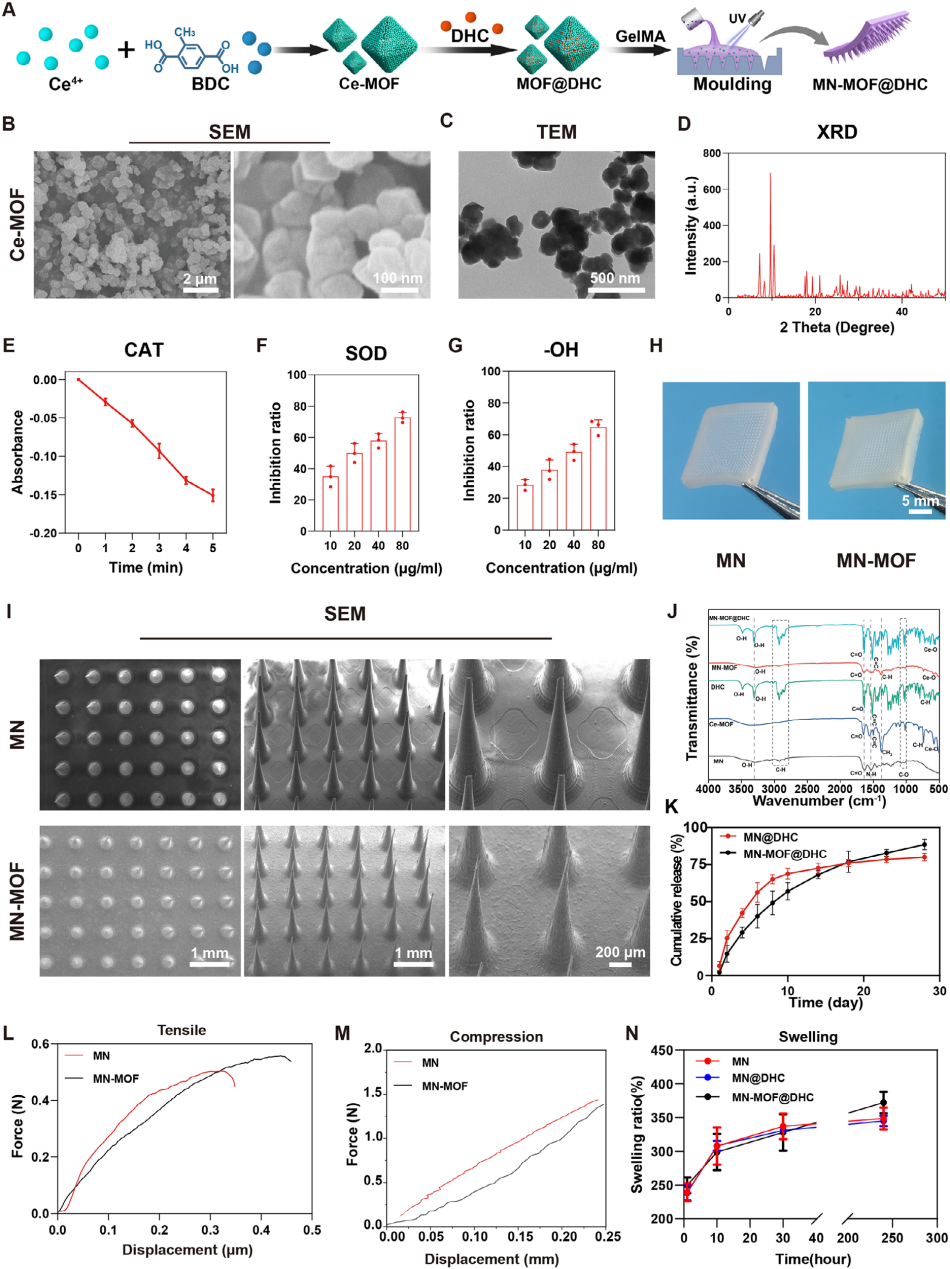
Synthesis and characterization of MN‐MOF@DHC. (A) Schematic diagram of preparation of MN‐MOF@DHC. (B) SEM images of Ce‐MOF. (C) TEM images of Ce‐MOF. (D) XRD of Ce‐MOF. (E–G) CAT‐mimicking, SOD‐mimicking, and·OH‐scavenging ability of Ce‐MOF. (H) Gross morphology of MN and MN‐MOF. (I) SEM images of MN and MN‐MOF. (J) FTIR of MN, Ce‐MOF, DHC, MN‐MOF, and MN‐MOF@DHC. (K) Dihydrocapsaicin (DHC) release curve of microneedles following incubation in a saline solution. (L–N) Tensile curve, compressive curve, and swelling ratio of the microneedles. (n = 3, error bars, means ± SD; all analyses were done using one‐way ANOVA with Tukey's post hoc test *p < 0.05, **p < 0.01, ***p < 0.001, and **** *P* < 0.0001).

On the basis of a previous research [47], we synthesized Ce‐MOFs loaded with DHC (Figure 1A). Scanning electron microscopy (SEM) and transmission electron microscopy (TEM) images revealed the morphology of the Ce‐MOF (Figure 1B‐C). The X‐ray diffraction (XRD) patterns obtained for the Ce‐MOFs were in agreement with the patterns previously reported in the literature [51], indicating the successful synthesis of the materials. (Figure 1D). The Ce^3+^ site facilitates the removal of superoxide radicals (O_2_^−^) through SOD‐like activity and generates hydroxyl radical (·OH) via redox reactions, the Ce^4+^ site is capable of decomposing H_2_O_2_ through CAT‐like activity in contrast [56, 57]. The CAT‐like activity of the Ce‐MOF was assessed by observing the absorbance change of H_2_O_2_ upon reaction with Ti(SO_4_)_2_ at a wavelength of 405 nm. A reduction in absorbance at this wavelength served as an indicator of H_2_O_2_ decomposition. The Ce‐MOF demonstrated notable CAT‐like activity (Figure 1E). Moreover, the SOD‐mimetic and ·OH‐scavenging abilities were assessed using commercially available test kits. For SOD‐mimetic activity, the xanthine (X)/xanthine oxidase (XO) system was used to produce O_2_^−^. These superoxide radicals possess the capability to transform nitro blue tetrazolium (NBT) into blue‐colored formazan, a compound that can be quantitatively measured at a wavelength of 560 nm. SOD activity was measured on the basis of the ability of Ce‐MOF to scavenge O_2_^−^ radicals. The OH‐scavenging ability was determined via the Fenton reaction, where the content of ·OH generated was quantified via the Griess reagent after the administration of an electron acceptor. Higher concentrations of MOFs increased the ability to scavenge O_2_^−^ radicals, indicating concentration‐dependent activity (Figure 1F‐G). Subsequently, digital photographs of both the standard MN and MN‐MOF were presented (Figure 1H). SEM images revealed the morphological characteristics of the microneedles. The dimensions of each microneedle were as follows: a base width of 300 µm, a needle spacing of 700 µm, and a height of 800 µm. Additionally, the average Ce‐MOF particle size was determined as 85.93±18.14 nm via Dynamic Light Scattering (DLS) (Figure S1). SEM images also indicated that MOFs were successfully incorporated into the GelMA microneedles: granular MOFs could be observed on the surface of the microneedles in the MN‐MOF group (Figure 1I). The increased surface roughness of the MN‐MOF material increases its suitability for cell adhesion and proliferation [58]. The corresponding zeta potential measurements (Figure S2) show that the surface negative charge of MN decreases after the addition of MOF@DHC, confirming the successful loading of positively charged MOF@DHC onto MN. All microneedles were analyzed by FTIR based on the chemical bond information (Figure 1J). In the FTIR spectra of MN‐MOF, the C = O related stretching vibration at 1633 cm^−^^1^ was characteristic of the amide group in GelMA and the organic ligand in MOF, proving that MN‐MOF was successfully prepared. In the FTIR spectra of MN‐MOF@DHC, all the characteristic peaks corresponding to MN‐MOF were observed in the composite nanomaterials. Additionally, the introduction of the DHC modification resulted in the incorporation of novel functional groups. The abundance of methyl and methylene groups in the DHC molecules led to an enhancement in the peak intensity of the C‐H stretching vibration region in the synthesized microneedles. After successfully fabricating MN‐MOF@DHC, we conducted several tests to assess its physicochemical properties. As shown in Figure 1K, the cumulative release of DHC from MN‐MOF@DHC was significantly greater than that from the microneedles alone, with the cumulative release time extending nearly 28 days (Figure 1K). The sustained release profile may be due to the presence of catalytic centers at the molecular level, the high porosity, and the significant loading capacity of Ce‐MOFs [51]. To further validate the degradation behavior of MN‐MOF@DHC in collagenase‐2 solution, we conducted degeneration testing and observed progressive degradation via SEM at different points. The MN‐MOF@DHC exhibited rapid yet controlled degradation, achieving complete dissolution at Day 16–20 (Figure S3). The SEM imaging suggested progressive degradation in microneedle tips characterized by morphological changes, including tip flattening and even disappearing over time. However, the bottom of the microneedles still remained nearly intact at Day 8 (Figure S4).

Previous investigations have frequently employed multiple drugs or bioactive factors for sequential release to address the diverse requirements of diabetic wound healing at various stages [59, 60, 61]. Nonetheless, the sequential biological process is severely disordered under the condition of persistent hyperglycemia, leading to intricately overlapping phases of inflammation, proliferation, and remodeling [8, 62, 63]. This complexity complicates the precise targeting of therapeutic interventions tailored to each specific stage. Consequently, the selection of a drug that exhibits prolonged stability and can concurrently improve every phase of wound healing becomes particularly critical in the whole‐course management of diabetic wounds. Mechanical testing revealed improved tensile and compressive properties for MN‐MOF compared with those of standard microneedles (Figure 1L,M). The ability of MN‐MOF to withstand greater mechanical pressure is particularly advantageous in wound healing. When applied to the wound site, the microneedles must maintain their structure during insertion into the skin and throughout the period they are required to deliver therapeutic agents or support tissue regeneration. Superior tensile strength ensures that the needles do not break off within the skin, thereby reducing the risk of infection and ensuring consistent delivery of the active components [64]. Furthermore, the notably higher swelling ratio suggests that MN‐MOF@DHC has the capability to efficiently absorb exudate from wound tissue, owing to the distinctive design of the microneedles (Figure 1N). We also conducted a microneedle‐insertion test on rat skin to validate the penetration capacity, both macroscopic and H&E staining evidence collectively supports its consistent transdermal penetration in rat skin (Figure S5).

Biocompatibility is a critical consideration when designing appropriate biomaterials for wound healing [65]. Firstly, we conducted a cellular uptake assay using fluorescence‐labeled Ce‐MOF (TRITC‐conjugated) and analyzed uptake efficiency in target cells via confocal microscopy (Figure S6). Based on prior literature and our CCK‐8 assay results (Figure S7), a DHC concentration of ​​100 nM was selected for in vitro cell studies to ensure optimal bioactivity while mitigating cytotoxicity [26]. For in vivo animal trials, to standardize DHC content between MN‐DHC (free drug) and MN‐MOF@DHC groups, the total DHC mass per microneedle patch was precisely controlled: for MN‐DHC, a defined DHC solution was directly incorporated into the polymer matrix, achieving the final concentration of 100 nM in the mixture. While for MN‐MOF@DHC, the DHC‐loaded MOF powder was dispersed such that the cumulative DHC mass matched that of the free drug group according to the loading efficiency of DHC in the Ce‐MOF. This approach ensures comparable therapeutic dosing for fair efficacy evaluation. The MN‐MOF@DHC exhibited excellent biocompatibility, attributed to its components, GelMA and MOF. To evaluate cell proliferation and cytotoxicity, we employed CCK‐8 assays and live/dead staining. As shown in Figure S1C, treatment with MN‐MOF, DHC, or MN‐MOF@DHC showed no impact on cell proliferation following 1, 3, and 5 days of observation, suggesting that these treatments did not have any detrimental effects on cell growth. Live/dead staining (Figure S8A, B) further confirmed that MN‐MOF@DHC maintained good biocompatibility, with no significant differences observed between MN‐MOF, DHC, or MN‐MOF@DHC groups and the control group. In vivo, MN‐MOF and DHC treatment did not induce observable pathological changes or tissue damage in major organs, indicating a favorable safety profile for the microneedles (Figure S9). These results collectively show the biocompatible nature of MN‐MOF@DHC, supporting its potential for use in wound healing applications.

### 3.2 Anti‐Oxidative and Anti‐Inflammatory Effects of MN‐MOF@DHC

**FIGURE 2:**
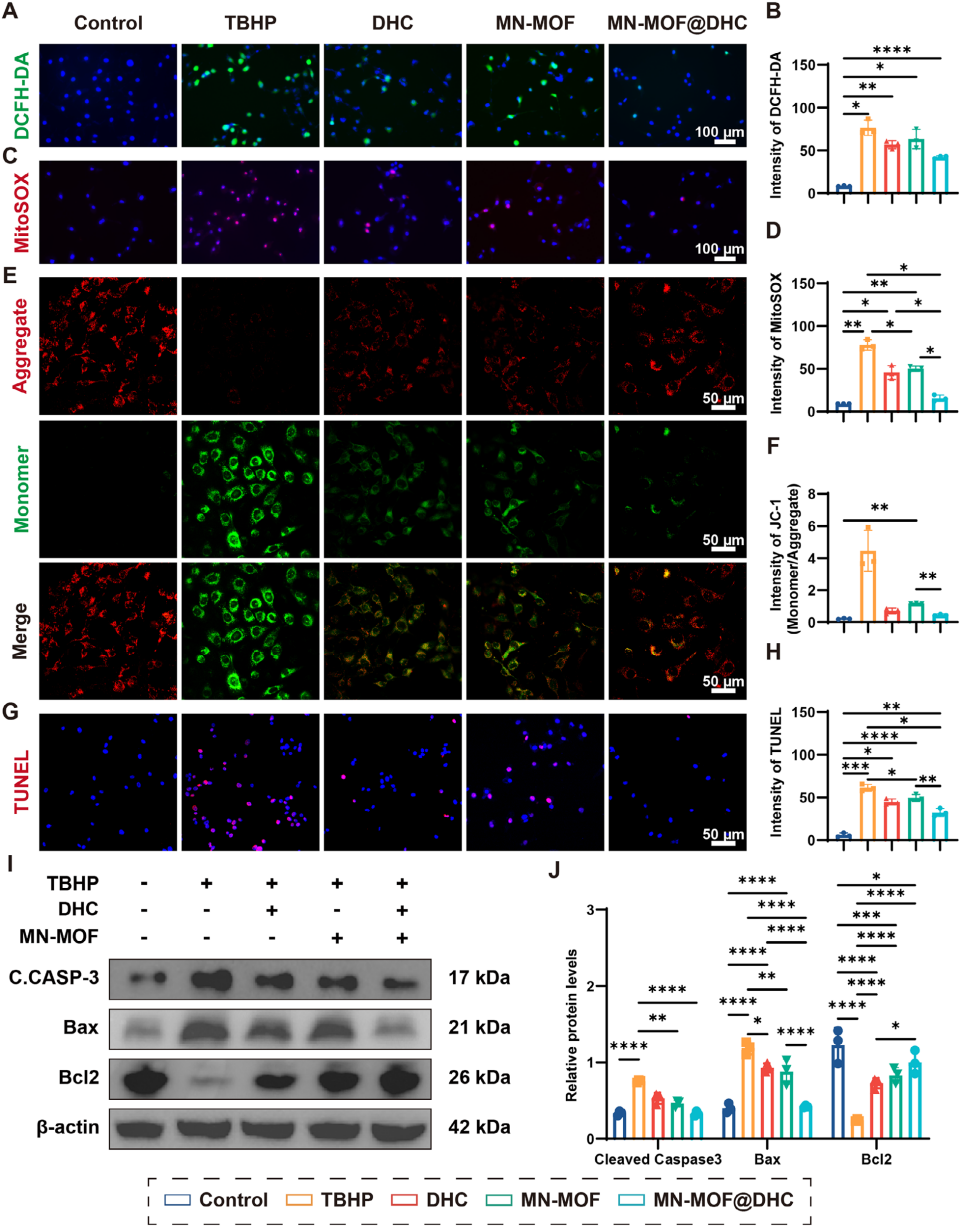
Assessment of the anti‐oxidant, anti‐apoptosis properties of MN‐MOF@DHC in vitro. (A, C) Images of HUVECs were obtained after staining with DCFH‐DA and MitoSOX, captured with diverse experimental conditions. (B, D) Semiquantitative analysis of fluorescence intensity. (E) Mitochondrial membrane potential was assessed by JC‐1 assay in HUVECs under diverse experimental conditions. (F) Semiquantitative analysis of JC‐1 fluorescence intensity. (G) TUNEL staining of HUVECs under different conditions. (H) Semiquantitative analysis of TUNEL staining. (I–J) The protein levels of cleaved caspase‐3 (C.CASP‐3), Bax, and Bcl‐2 were measured by western blotting and semiquantitative evaluation. (n = 3, error bars, means ± SD; all analyses were done using one‐way ANOVA with Tukey's post hoc test *p < 0.05, **p < 0.01, ***p < 0.001, and **** *P* < 0.0001).

The inflammatory response accompanying the development of chronic diabetic wounds has been extensively studied to improve patient outcomes [66, 67, 68]. Previous studies have shown that ROS activate the NF‐κB pathway, which initiates pro‐inflammatory responses by enhancing the phosphorylation of I‐κB, the inhibitor of NF‐κB. Consequently, the antioxidant capabilities of hydrogels are vital in modulating inflammation in diabetic wounds [29]. To assess the levels of intracellular and mitochondrial ROS in HUVECs, DCFH‐DA and MitoSOX probes were employed. The microneedles exhibited outstanding catalytic activity, as indicated by the steady reduction in fluorescence intensity observed in DCFH‐DA‐stained cells (Figure 2A,B) and MitoSOX‐labeled cells (Figure 2C,D). Notably, compared with MN‐MOF or DHC alone, MN‐MOF@DHC exhibited superior anti‐ROS efficacy. Oxidative stress can induce mitochondrial structural damage, consequently disrupting mitochondrial membrane potential and resulting in cell death [69]. Alterations in the mitochondrial membrane potential of HUVECs were assessed utilizing JC‐1 fluorescent probes. TBHP‐treated HUVECs shifted from aggregate (red) to monomer (green) fluorescence, indicating depolarization. However, coculture with MN‐MOF@DHC resulted in decreased green fluorescence intensity, suggesting reduced mitochondrial membrane potential depolarization (Figure 2E). Subsequent semi‐quantification confirmed that the synergistic effect of DHC and Ce‐MOF markedly alleviated oxidative stress damage (Figure 2F). TUNEL staining and western blot results further revealed that exposure to TBHP accelerated apoptosis in HUVECs, while treatment with DHC, MN‐MOF, or MN‐MOF@DHC significantly mitigated this apoptotic effect (Figure 2G–J). Notably, MN‐MOF@DHC demonstrated the most pronounced ability to mitigate cellular apoptosis.

**FIGURE 3:**
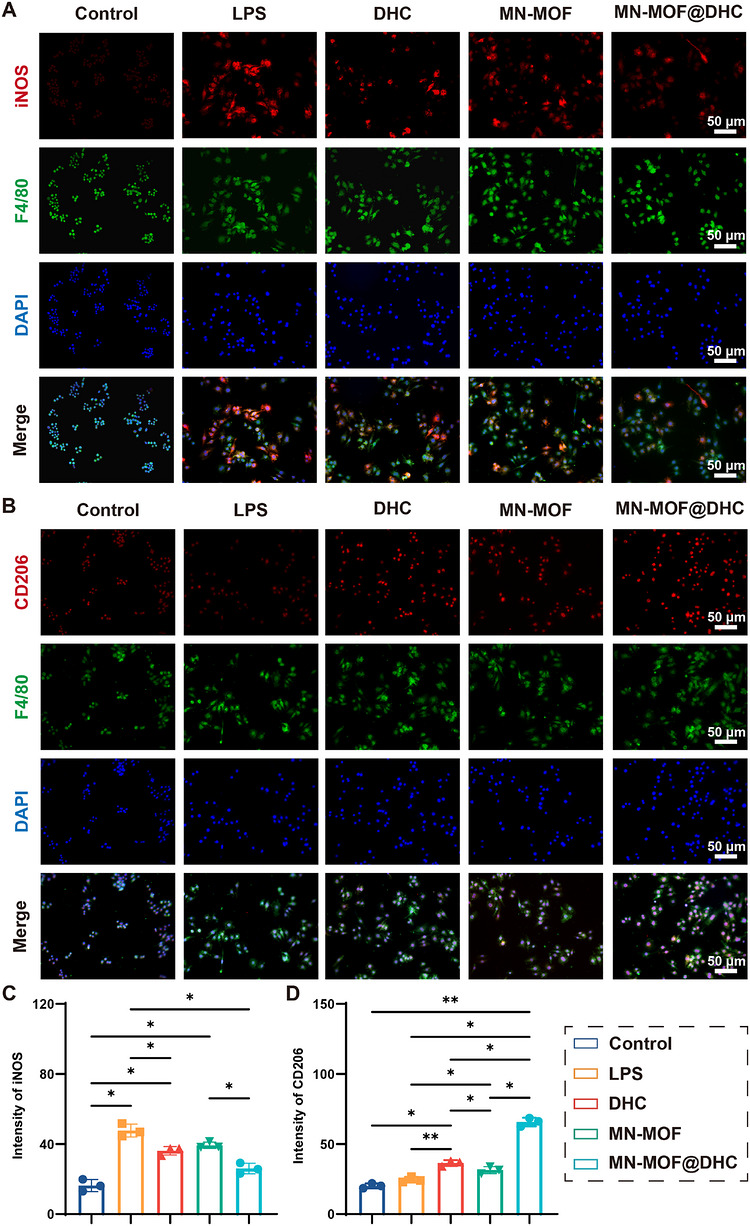
Regulation of macrophage phenotypes with MN‐MOF@DHC. (A) Immunofluorescence images of iNOS expression in macrophages cocultured with DHC, MN‐MOF, and MN‐MOF@DHC. (B) Immunofluorescent images of CD206 expression in macrophages cocultured with DHC, MN‐MOF, and MN‐MOF@DHC. (C) semiquantitative assessment of iNOS in macrophages. (D) semiquantitative assessment of CD206 in macrophages. (n = 3, error bars, means ± SD; all analyses were done using one‐way ANOVA with Tukey's post hoc test *p < 0.05, **p < 0.01, ***p < 0.001, and **** *P* < 0.0001).

We further then co‐cultured macrophages with DHC, MN‐MOF, and MN‐MOF@DHC to observe alterations in their polarization status. Immunofluorescence analysis showed a significant reduction in the expression level of the M1 marker, iNOS, indicating reduced M1 macrophage polarization (Figure 3A,C). Additionally, immunofluorescence demonstrated that MN‐MOF@DHC promoted the expression of the M2 marker CD206 (Figure 3B, D). Besides, enzyme‐linked immunosorbent assay (ELISA) results confirm a distinct increase in IL‐4, IL‐10, and IL‐13 in MN‐MOF@DHC‐treated sample compared to controls (Figure S10), and polymerase chain reaction (PCR) results (Figure S11) showed that MN‐MOF@DHC treatment notably upregulated Arg1, as well as the expression of a typical M2 marker, Mrc1. These findings confirm that the fabricated microneedles have the capacity to facilitate the transition of proinflammatory M1 macrophages into anti‐inflammatory M2 macrophages. The transition from M1 to M2 macrophage polarization observed following MN‐MOF@DHC treatment underscores the potential therapeutic advantages of this system in regulating the immune microenvironment during the wound healing process. The M1 phenotype is typically associated with a pro‐inflammatory state that can exacerbate tissue damage and delay healing, particularly in chronic wounds such as those seen in diabetic patients. Conversely, the M2 phenotype is characterized by its anti‐inflammatory properties and its ability to promote tissue repair and angiogenesis [70, 71].

In this study, the enhanced performance of MN‐MOF@DHC is attributed to the synergistic effect between Ce‐MOF (which scavenges ROS and provides a protective microenvironment) and DHC (which exerts anti‐inflammatory effects). The sustained release and improved stability of DHC loaded in MOFs further contribute to the enhanced efficacy. This enhanced efficacy may be attributable to two interconnected mechanisms. On the one hand, the porous architecture of Ce‐MOF shields encapsulated DHC from enzymatic breakdown, enabling sustained bioactivity over extended durations. On the other hand, DHC stabilizes the Ce^3^^+^/Ce^4^^+^ redox centers within the MOF framework, preventing catalytic site deactivation and amplifying ROS‐scavenging kinetics [26, 48, 72]. These complementary mechanisms operate concertedly to potentiate the overall antioxidant response, demonstrating a biomaterial‐drug synergy critical for diabetic wound remediation.

### 3.3 MN‐MOF@DHC Enhances Cell Migration and Angiogenesis in Vitro

In the treatment of chronic diabetic wounds, enhanced cell migration and accelerated vascular regeneration serve as crucial factors in facilitating wound healing [73, 74]. Enhanced cell migration promotes wound closure by enabling cells to actively traverse into and populate the injured tissue, thereby facilitating tissue repair. Meanwhile, vascular regeneration plays a pivotal role by fostering the development of new blood vessels, which are indispensable for supplying vital nutrients and oxygen to the healing site, thereby supporting and accelerating the overall healing process. Together, these processes play critical roles in overcoming the delayed healing often associated with diabetic wounds [12]. Given that DHC can effectively promote angiogenesis [24], we loaded DHC into the MOF during the preparation of the microneedle material, allowing for its gradual and sustained release into the tissue environment. The release tests indicated that DHC was gradually released from the MN‐MOF over a period of approximately 28 days (Figure 1K).

**FIGURE 4:**
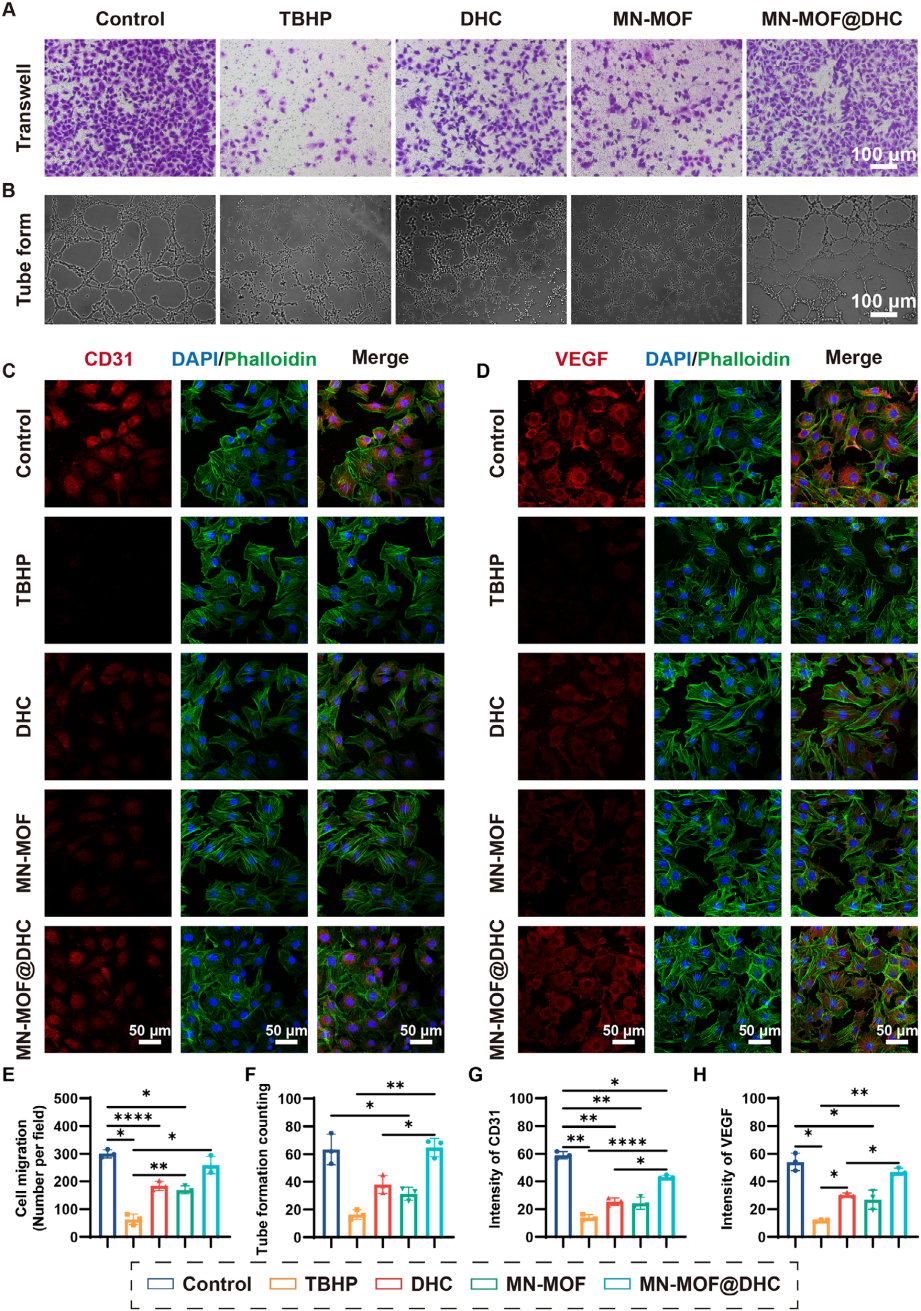
In vitro assessment of the angiogenesis properties of MN‐MOF@DHC. (A) Transwell migration of HUVECs under various conditions. (B) Tube formation of HUVECs under various conditions. (C,D) Immunofluorescent images of CD31 and VEGF in HUVECs cocultured with various conditions. (E,F) Statistical evaluation of transwell migration and tube formation. (G‐H) Semiquantitative analysis of CD31 and VEGF fluorescence intensity. (n = 3, error bars, means ± SD; all analyses were done using one‐way ANOVA with Tukey's post hoc test *p < 0.05, **p < 0.01, ***p < 0.001, and **** *P* < 0.0001).

Migration assays for HUVECs verified that the DHC released from the microneedles had a significant stimulatory effect on cell migration. Specifically, in the MN‐MOF@DHC‐treated group, the rate of cell migration reached 64.7% ± 5.4% after 12 h (Figure 4A,E). Tube formation assays further confirmed that MN‐MOF@DHC improved the ability of HUVECs to form tubular structures in controlled in vitro conditions (Figure 4B,F). The sustained release of DHC from MN‐MOF@DHC led to a significant upregulation of CD31 and VEGF expression in HUVECs when cocultured with the microneedles (Figure 4C,D). Subsequent semiquantitative analysis of immunofluorescence for both CD31 and VEGF confirmed the protective effect on HUVECs (Figures 4G, H). In conclusion, these findings suggested that MN‐MOF@DHC has the potential to accelerate the healing process of chronic diabetic wounds by enhancing cell migration and stimulating vascular regeneration, thus creating a favorable environment for wound repair.

### 3.4 MN‐MOF@DHC Accelerates the Healing of Diabetic Wounds in Vivo

**FIGURE 5:**
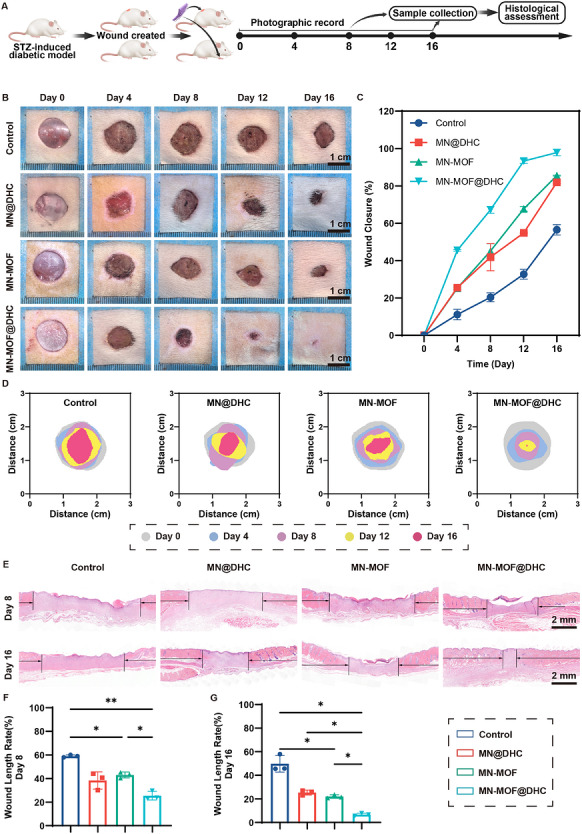
MN‐MOF@DHC enhanced wound closure in diabetic rats. (A) Schematic diagram of the application process of therapeutic microneedles in animal experiments. (B) Illustrative images showed the diabetic wounds across various treatment groups. (C) Measurement and quantitative analysis of the residual wound areas were conducted on day 4, 8, 12, and 16 post‐treatment for the various groups. (D) Traces of the healing process of diabetic wounds under various treatment groups. (E) Histological examination through H&E staining of the wound tissue in various groups on days 8 and 16. (F‐G) Quantitative analysis of wound length rate on days 8 and 16. (n = 3, error bars, means ± SD; all analyses were done using one‐way ANOVA with Tukey's post hoc test *p < 0.05, **p < 0.01, ***p < 0.001, and **** *P* < 0.0001). .

As shown in Figure 5A, diabetes was induced in rats using a high‐fat, high‐sugar diet followed by an intraperitoneal injection of streptozotocin (STZ) [75]. Once diabetes was successfully induced, wounds of a 15 mm diameter were created in a circular shape on the dorsal region of the diabetic rats. Post‐surgery, the wounds were treated with either MN‐MOF loaded with DHC (MN‐MOF@DHC) or without DHC (MN‐MOF). Additionally, in the MN@DHC group, microneedles loaded with DHC without Ce‐MOF were put into the wound area. Digital photographs of the wounds indicated that the MN‐MOF@DHC group achieved the best wound healing rate, with nearly 90% closure by day 12 and almost complete healing by day 16 (Figure 5B,D). Experimental data pertaining to wound closure rates provided evidence that MN‐MOF@DHC effectively accelerated the healing process of chronic wounds in diabetic rats (Figure 5C). Subsequent H&E staining of the wound tissue sections at days 8 and 16 demonstrated the smallest wound areas in the MN‐MOF@DHC‐treated group (Figure 5E). Quantitative analysis of wound length distinctly revealed the enhanced efficacy of MN‐MOF@DHC in facilitating the healing of chronic wounds in diabetic rats (Figure 5F,G).

The superior performance of the MN‐MOF@DHC compared to the MN@DHC group underscores the advantage of microneedle‐mediated transdermal delivery in achieving sustained local drug concentration while avoiding systemic side effects, a limitation frequently reported in oral antioxidant therapies [76]. Also, the accelerated wound closure observed in the MN‐MOF@DHC group aligns with emerging evidence that localized drug delivery systems targeting oxidative stress and inflammation significantly improve diabetic wound healing outcomes [77, 78, 79, 80, 81]. The local microenvironment of diabetic ulcers is characterized by an inflammatory‐regenerative imbalance, resulting in prolonged healing times of about 7–8 days compared to normal wounds, with full‐thickness skin defect wound closure typically requiring 21–28 days. Therefore, our microneedle sustained‐release system, capable of maintaining drug release for 28 days, ensures therapeutic efficacy throughout the entire healing process of diabetic ulcers, addressing a critical unmet need in chronic wound management [82]. Chen et al. synthesized a nanozyme, PDA‐MnO_2_ (PM), which was encapsulated in the needle tip layer of a bi‐layer GelMA microneedle patch (PbMP). This PbMP exhibits excellent mechanical properties, allowing it to easily penetrate the skin and deliver therapeutic agents directly to the wound site. The PbMP significantly accelerated the healing process of bacterially challenged wounds, resulting in superior blood vessel growth, cell proliferation, and collagen biosynthesis compared to other wound dressings [83]. Yang et al. have introduced a multifunctional microneedle patch loaded with Yunnan Baiyao, designated as (BY + EGF)@MN, and confirmed its application in rat cutaneous wounds. Their findings indicate that this patch can expedite the wound healing process by augmenting neovascularization, increasing fibroblast density, and promoting collagen deposition [84].

### 3.5 MN‐MOF@DHC Attenuates Oxidative Stress, Regulates Macrophage Polarization, and Inhibits Apoptosis in Vivo

**FIGURE 6:**
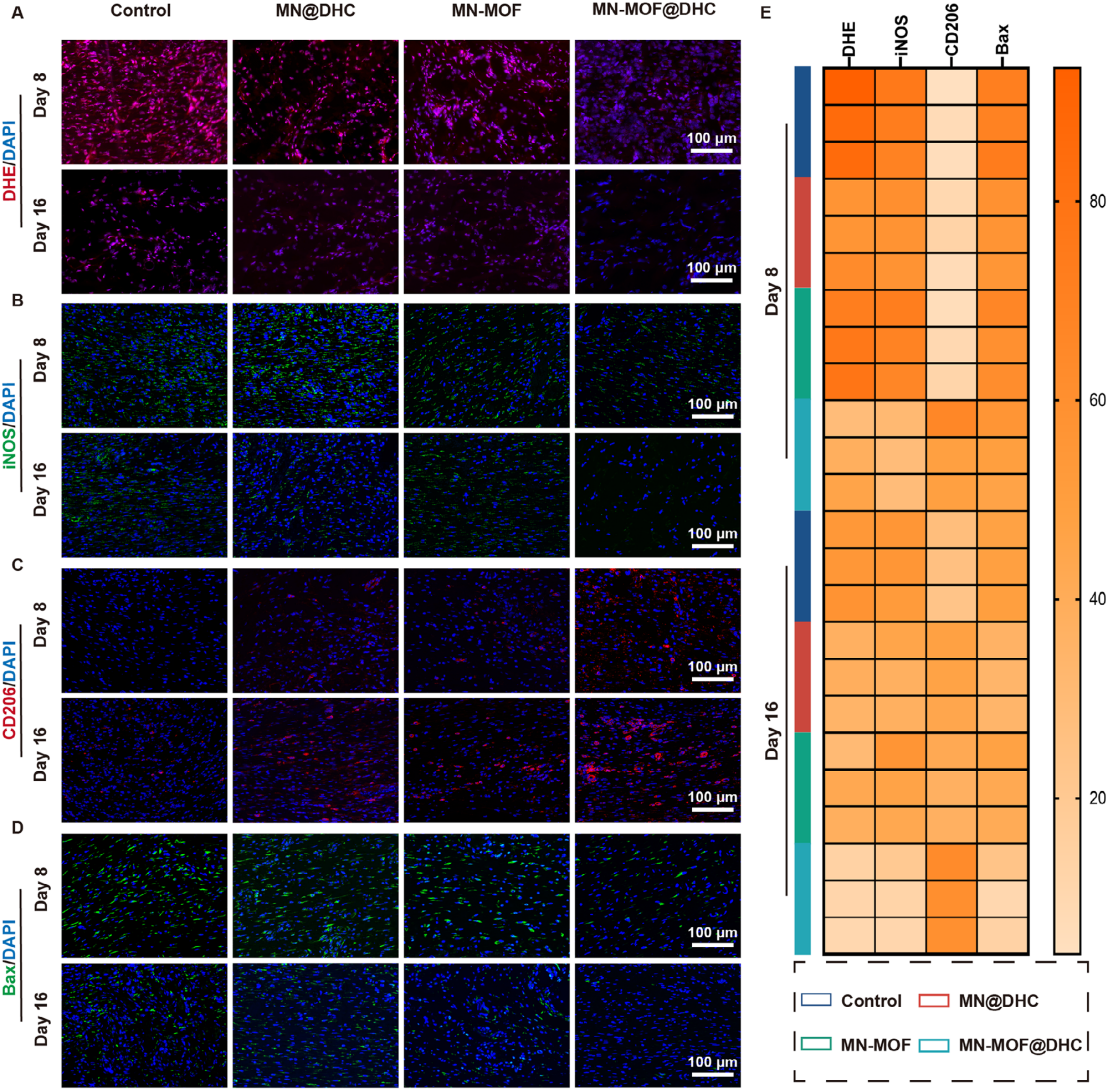
MN‐MOF@DHC reduced oxidative stress, inflammation, and apoptosis in diabetic wound tissues. (A) Immunofluorescence images of wound tissue sections from a diverse group following DHE staining on days 8 and 16. (B‐C) Immunofluorescence staining images of iNOS and CD206 captured on days 8 and 16. (D) Immunofluorescence images of Bax on days 8 and 16. (E) Semiquantitative analysis of DHE, iNOS, CD206, and Bax fluorescence intensity on days 8 and 16. (n = 3, error bars, means ± SD; all analyses were done using one‐way ANOVA with Tukey's post hoc test *p < 0.05, **p < 0.01, ***p < 0.001, and **** *P* < 0.0001).

In diabetic wounds, persistent high blood sugar levels result in excessive production of ROS and subsequent oxidative stress, serving as key factors that hinder the wound healing process [19, 85, 86]. Excessive ROS maintains the local wound environment in a state of chronic inflammation, impairing endothelial cell function and ultimately leading to nonhealing wounds [87, 88]. Therefore, removing excess ROS and regulating the local inflammatory microenvironment are vital for diabetic wound regeneration. To explore the effects of MN@DHC, MN‐MOF, and MN‐MOF@DHC on oxidative stress in diabetic wounds, we utilized DHE fluorescent probes to quantitatively measure ROS levels within wound tissues. This approach enabled us to assess the in vivo antioxidant activity of these treatments in the context of diabetic wound healing. On days 8 and 16, DHE staining of wound tissues revealed that both MN‐MOF and MN@DHC successfully scavenged ROS, with MN‐MOF@DHC demonstrating the most potent antioxidative effect (Figure 6A). Statistical analysis further confirmed these observations (Figure 6E). To gain a deeper understanding of the inflammatory microenvironment within the wound, we conducted immunofluorescence staining for iNOS and CD206 on tissue sections. The results shown in Figure 6B,C demonstrated that MN‐MOF@DHC demonstrated a significant inhibitory effect on inflammation within the wound tissues. Bax is a water‐soluble protein that has a similar structure to Bcl2 and plays a crucial role in triggering apoptosis within the Bcl2 gene family [89]. Immunofluorescence staining for Bax indicated that MN‐MOF@DHC effectively protected cellular function and inhibited cell apoptosis (Figure 6D). Overall, these findings highlight the potential capacity of the microneedles to attenuate oxidative stress, inhibit over‐productive and continuous inflammatory responses, and expedite the progression of chronic wounds into the healing phase. These effects underscore the therapeutic potential of MN‐MOF@DHC in promoting effective wound healing in diabetic wounds.

### 3.6 MN‐MOF@DHC Enhances the Regeneration of Blood Vessels in Vivo

**FIGURE 7:**
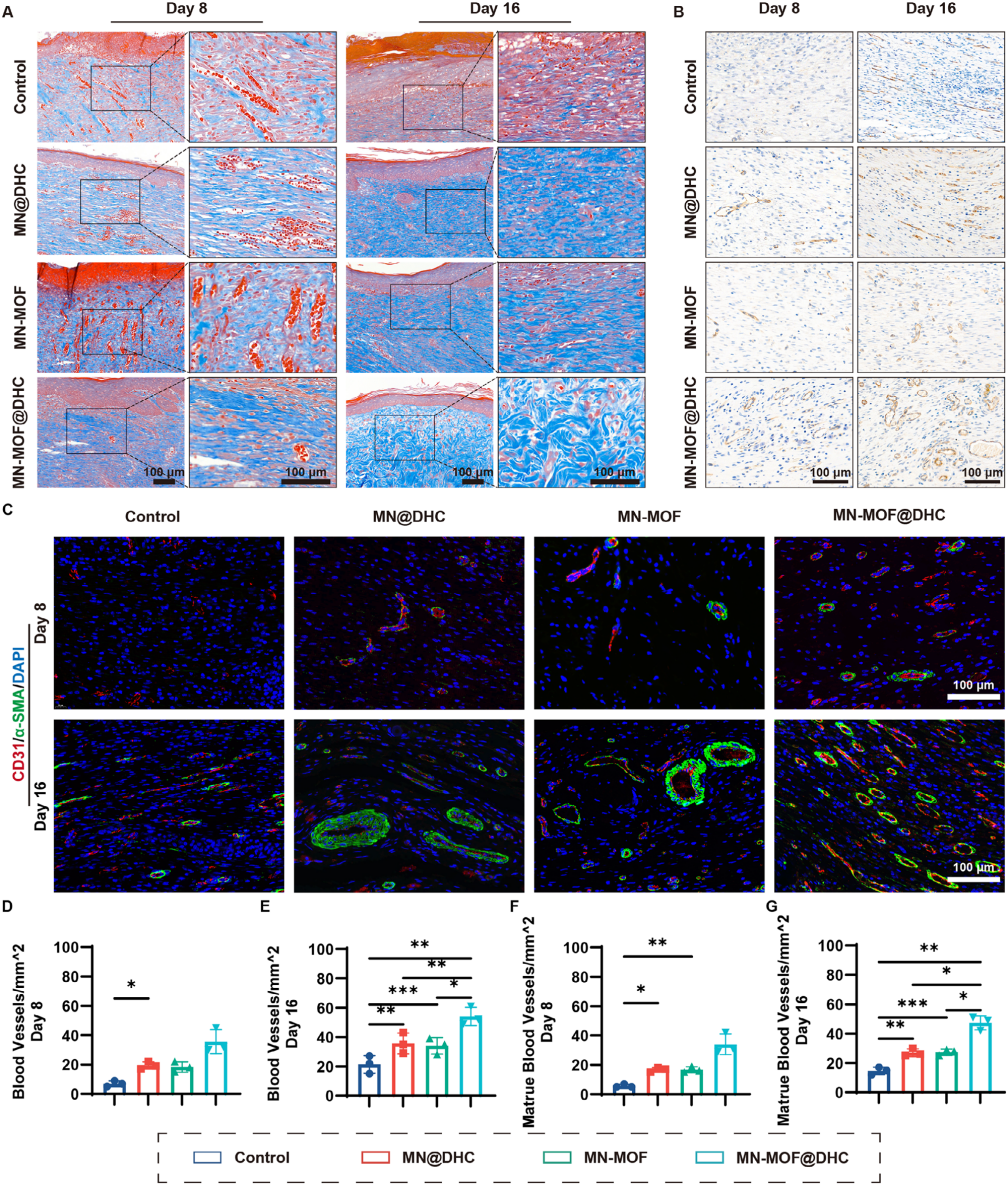
In vivo assessment of the angiogenic properties of MN‐MOF@DHC in a diabetic wound model. (A) Visual representation of Masson‐stained wounds on days 8 and 16 post‐treatment with MN@DHC, MN‐MOF, and MN‐MOF@DHC. (B) Immunohistochemical staining images depicting CD31 expression in wound tissues on days 8 and 16. (C) Immunofluorescence staining images for CD31 and α‐SMA in diabetic wound tissues on days 8 and 16. (D‐E) Quantitative analysis of newly formed blood vessels. (F‐G) Quantitative assessment of mature blood vessels. (n = 3, error bars, means ± SD; all analyses were done using one‐way ANOVA with Tukey's post hoc test *p < 0.05, **p < 0.01, ***p < 0.001, and **** *P* < 0.0001).

Furthermore, Masson's trichrome staining revealed more frequent deposition of collagen in the MN‐MOF@DHC group (Figure 7A), indicating enhanced tissue regeneration. Insufficient blood vessel formation represents a significant factor contributing to the prolongation of healing time in diabetic wounds [90, 91]. To investigate the angiogenic effects of MN‐MOF@DHC in vivo, wound tissues were harvested on days 8 and 16 and subjected to histological and immunofluorescence staining. The impact on angiogenesis was assessed through immunohistochemical analysis and double immunofluorescence staining targeting CD31 and α‐SMA. Immunohistochemical and double immunofluorescence staining (Figures 7B‐C) revealed a recovery of blood vessel density and number after treating with MN@DHC, MN‐MOF, or MN‐MOF@DHC, with the most pronounced effect observed in the MN‐MOF@DHC group. Quantification of total and mature vessel numbers further confirmed these findings, showing that MN‐MOF@DHC significantly increased both metrics compared with the other treatments (Figure 7D‐G). As demonstrated in Figure S12, immunofluorescence co‐staining assays revealed that diabetic wound tissues treated with MN‐MOF@DHC exhibited both higher VEGF and higher Ang‐1 expression compared to other groups. These findings showed that MN‐MOF@DHC synergistically upregulates angiogenesis through coordinated modulation of the VEGF/Ang‐1 signaling axis.

### 3.7 MN‐MOF@DHC Accelerates Wound Closure in a DFU Model

**FIGURE 8:**
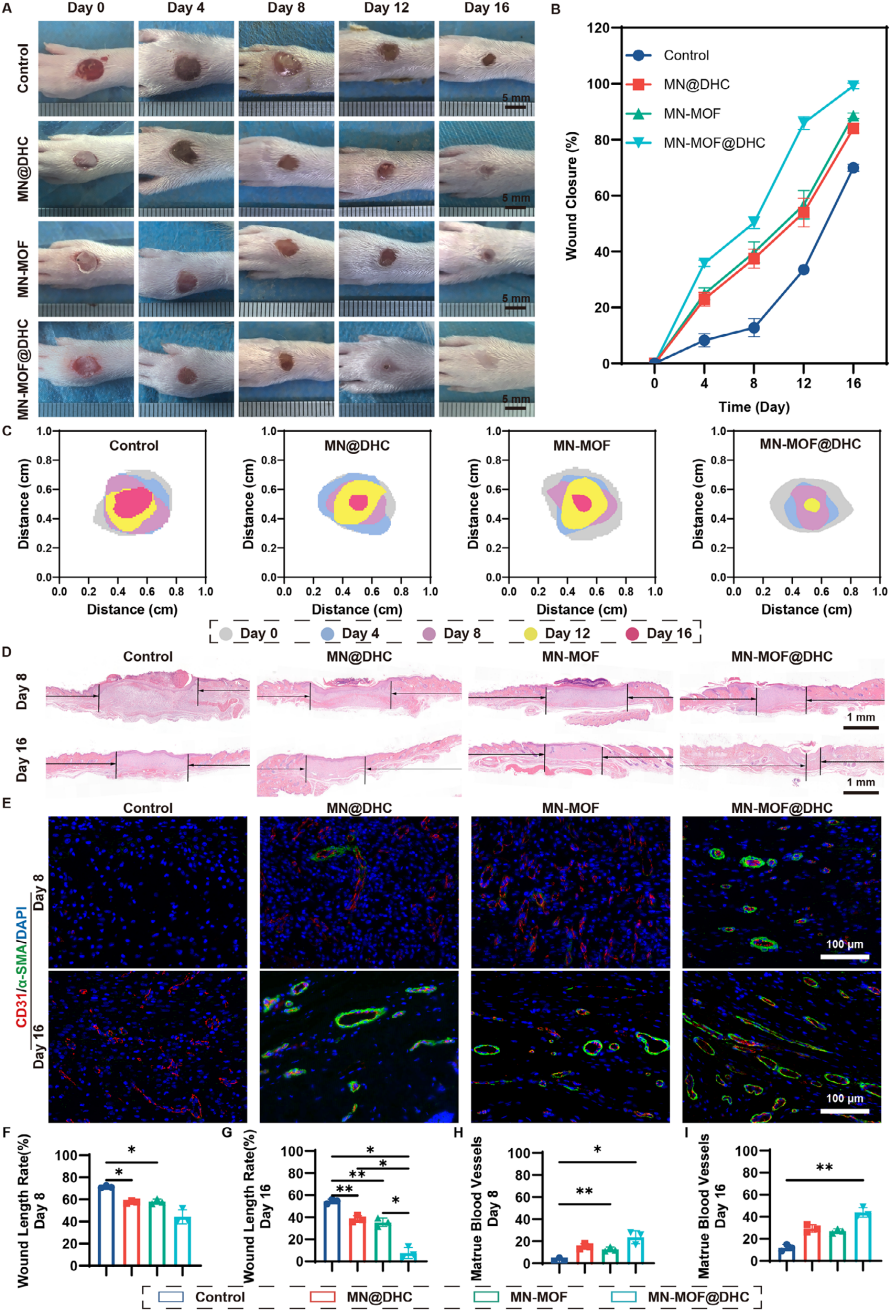
In vivo evaluation of MN‐MOF@DHC on a diabetic feet wound model. (A) Representative photographs of the diabetic feet wounds under various treatment groups. (B) Measurement of the residual areas of foot wounds following treatment administered by various groups. (C) Traces of diabetic feet wound healing processes under diverse treatment groups. (D) Histological examination through H&E staining of the diabetic feet wound areas in various groups on days 8 and 16. (E) Immunofluorescence images of CD31 and α‐SMA in feet diabetic wound tissues on days 8 and 16. (F‐G) Quantitative analysis of feet wound length rate on days 8 and 16. (H‐J) Quantitative measurement of mature blood vessels in feet wound tissues. (n = 3, error bars, means ± SD; all analyses were done using one‐way ANOVA with Tukey's post hoc test *p < 0.05, **p < 0.01, ***p < 0.001, and **** *P* < 0.0001).

To better simulate a real DFU model [92], we established a full‐thickness skin wound model in the feet of STZ‐treated diabetic rats (Figure 5A). Digital photographs and measurements of the wound closure rate indicated that the MN‐MOF@DHC group achieved the best wound healing outcomes, with wounds nearly completely healed by day 16 (Figure 8A‐C). Histological evaluation on days 8 and 16 further assessed the quality of wound healing (Figure 8D). Compared with the other groups, the MN‐MOF@DHC group clearly exhibited superior repair characteristics, including shorter wound lengths, more orderly granulation tissue formation, increased re‐epithelialization thickness, and faster morphological healing (Figures 8F‐G). Furthermore, double immunofluorescence staining targeting CD31 and α‐SMA demonstrated the reestablishment of mature blood vessels after administration of MN@DHC, MN‐MOF, or MN‐MOF@DHC, with the MN‐MOF@DHC group exhibiting the most pronounced effect (Figure 8E,H,I). These results demonstrate that MN‐MOF@DHC is a favorable microneedle formulation for DFU healing. The structure can continuously deliver DHC to the wound site, thereby enhancing cell proliferation, migration, and vascularization and accelerating the diabetic wound healing process.

## 4 Conclusion

The treatment of DFUs remains challenging because of the persistent high levels of oxidative stress and impaired vascular regeneration during the whole‐course management of diabetic wounds. Our research centered on creating an innovative solution by harnessing the distinctive characteristics of GelMA microneedles integrated with MOFs, further loaded with DHC to form MN‐MOF@DHC. The synthesized MN‐MOF@DHC exhibited significant mechanical strength and robust structural integrity for effective wound support; and it also enabled controlled local drug delivery, ensuring the sustained release of DHC for prolonged therapeutic effects while promoting a conducive environment for tissue repair.

A pivotal highlight of this microneedle formulation is its various therapeutic potential: through its strong antioxidant properties, it significantly reduces ROS levels, alleviating oxidative stress; by regulating inflammatory responses, it mitigates chronic inflammation that typically hinders wound healing; and by inhibiting apoptosis in endothelial cells, it enhances angiogenesis in the wound areas. Collectively, these coordinated responses facilitate the rapid healing of chronic wounds in diabetics. However, before these findings can be translated into clinical practice, several critical aspects require further investigation. Further research, encompassing clinical trials and process refinement, is required to confirm their effectiveness in human patients. Nonetheless, the development of MN‐MOF@DHC represents a significant step forward in addressing the challenges associated with the whole‐course diabetic wound management, paving the way for innovative solutions in regenerative medicine.

## Author Contributions

Yuxu Wu, Kan Jiang, and Xiao Wen were responsible for designing and conducting all the experiments, as well as drafting the manuscript. The data analysis was carried out by Chihao Jin, Kai Zhang, Jiakai Zhang, Jiale Jin, and Jiaqian Wang. The linguistic polishing of manuscript was performed by Huiyu Hu. Yihe Hu, Xianzhu Zhang, and Zonghan Xu contributed to the project design and provided review and editing of the manuscript. All authors have read and given their approval to the final manuscript.

## Funding

This project was supported by National Key Research and Development Program of China (No. 2023YFC2412604), the National Natural Science Foundation of China (No. 82272452, 82301016, 82403934), the Natural Science Foundation of Zhejiang Province (No. 2024C03079), the Natural Science Foundation of Jiangsu Province (No. BK20240383), and Gusu Health Talent Program (No. GSWS2024182).

## Ethics Statement

Rat: The Sprague‐Dawley (SD) rats (6 weeks old) were purchased from the Center of Experimental Animals of Zhejiang Province and housed at our hospital. All experimental procedures conformed to the standards for laboratory animal care and use, and were approved by the institutional review board of the First Affiliated Hospital, Zhejiang University School of Medicine. The approve number is 2025016.

## Conflicts of Interest

The authors declare that they have no competing interests.

## Supporting information




**Supporting File**: exp270176‐sup‐0001‐SuppMat.pdf.

## Data Availability

The corresponding author will provide all data used and/or analyzed in this study upon receipt of a reasonable request.
