# Supplementary material for: Sustained‐Release Ulcer‐Protective Microarray Patch With Dihydrocapsaicin for Diabetic Wound Regeneration
Source: Exploration (Beijing). 2026 May 28;6(4):20250129. doi: 10.1002/EXP.20250129 (PMC13394189; doi:10.1002/EXP.20250129)
Supplement: Supplementary file 1 [file EXP2-6-20250129-s001.pdf]

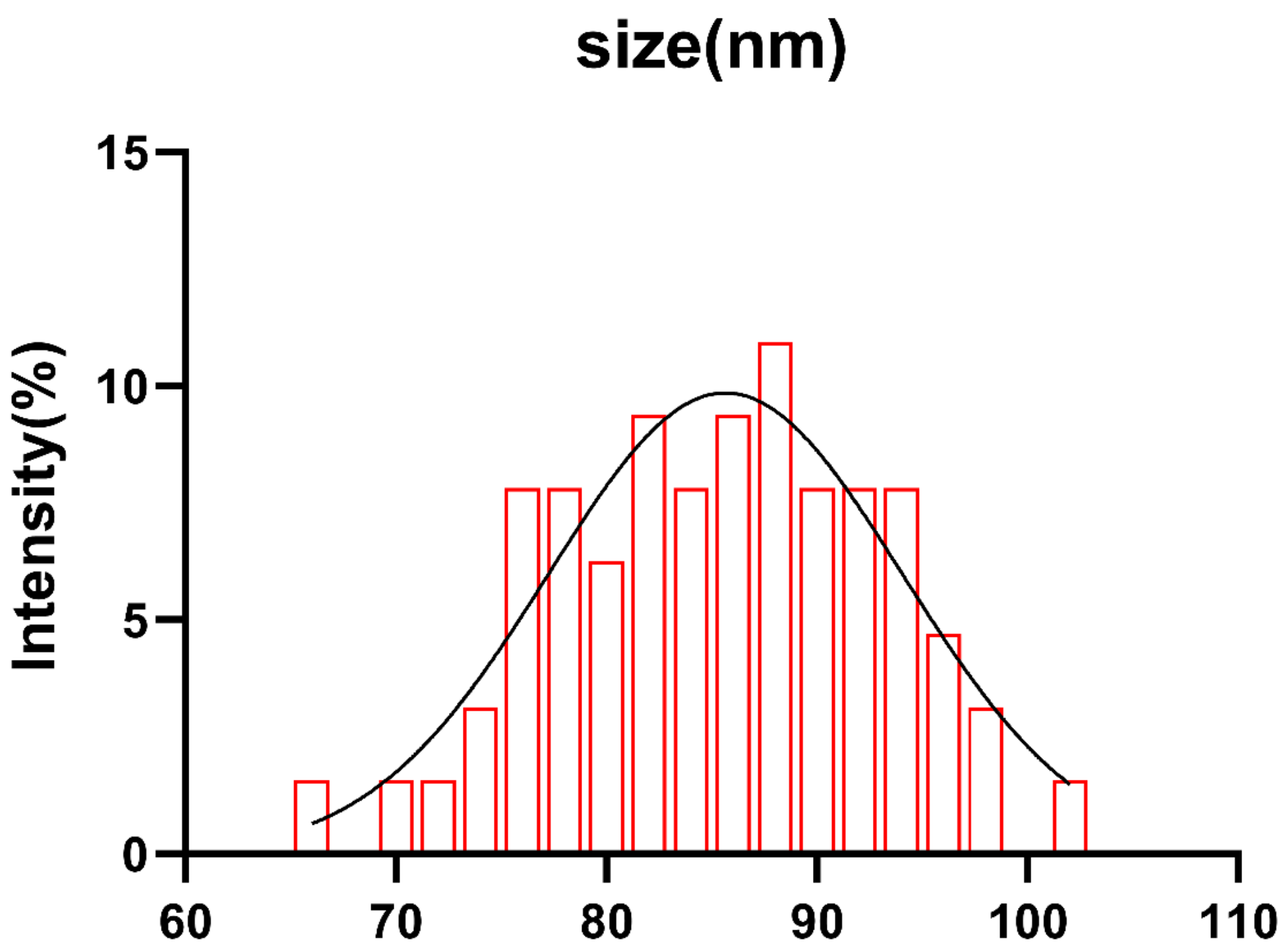

Supplementary Figure 1. The particle size of Ce-MOF.

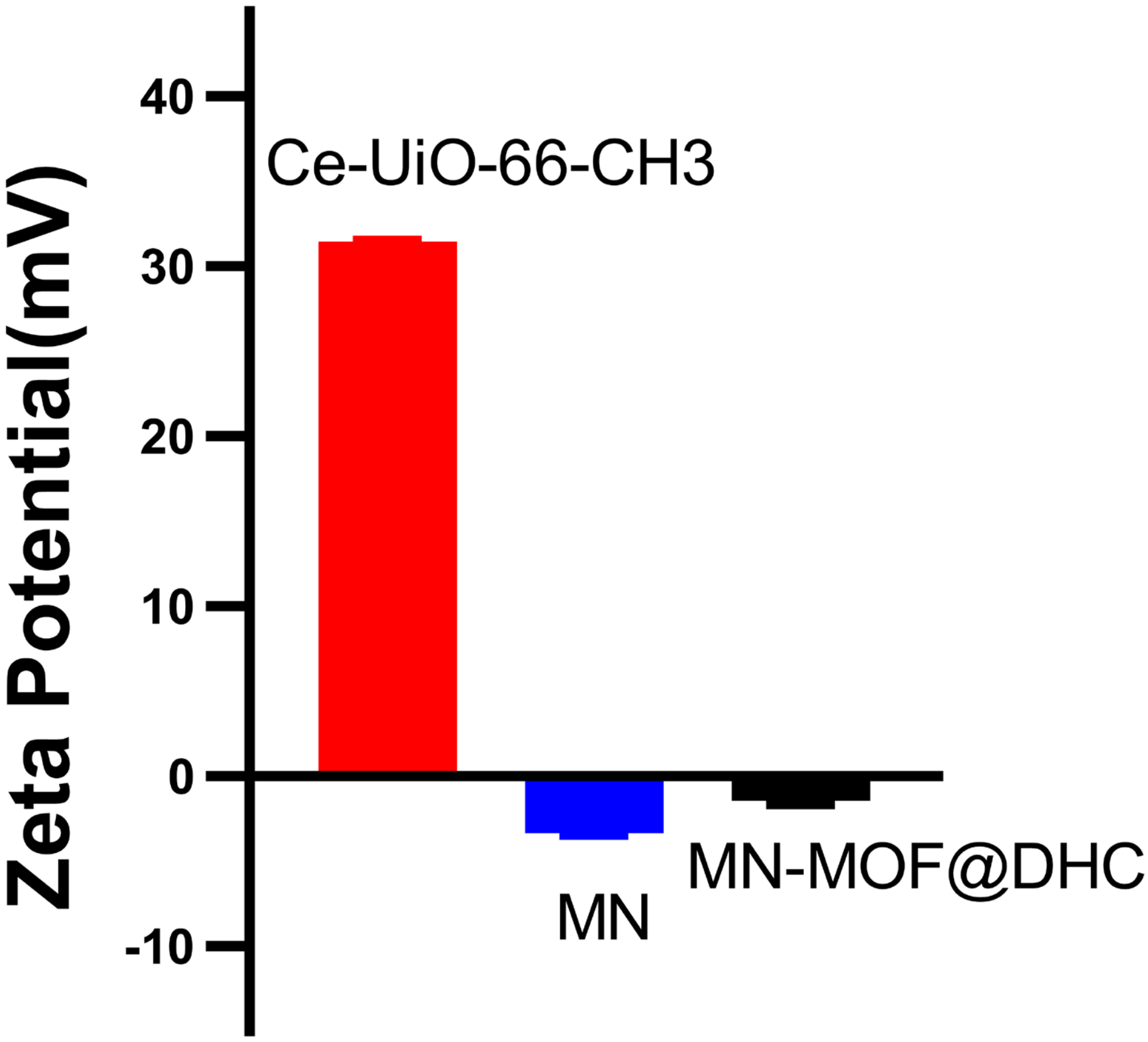

Supplementary Figure 2. The surface charge of nanoparticles was quantified utilizing a zeta potential analyzer.

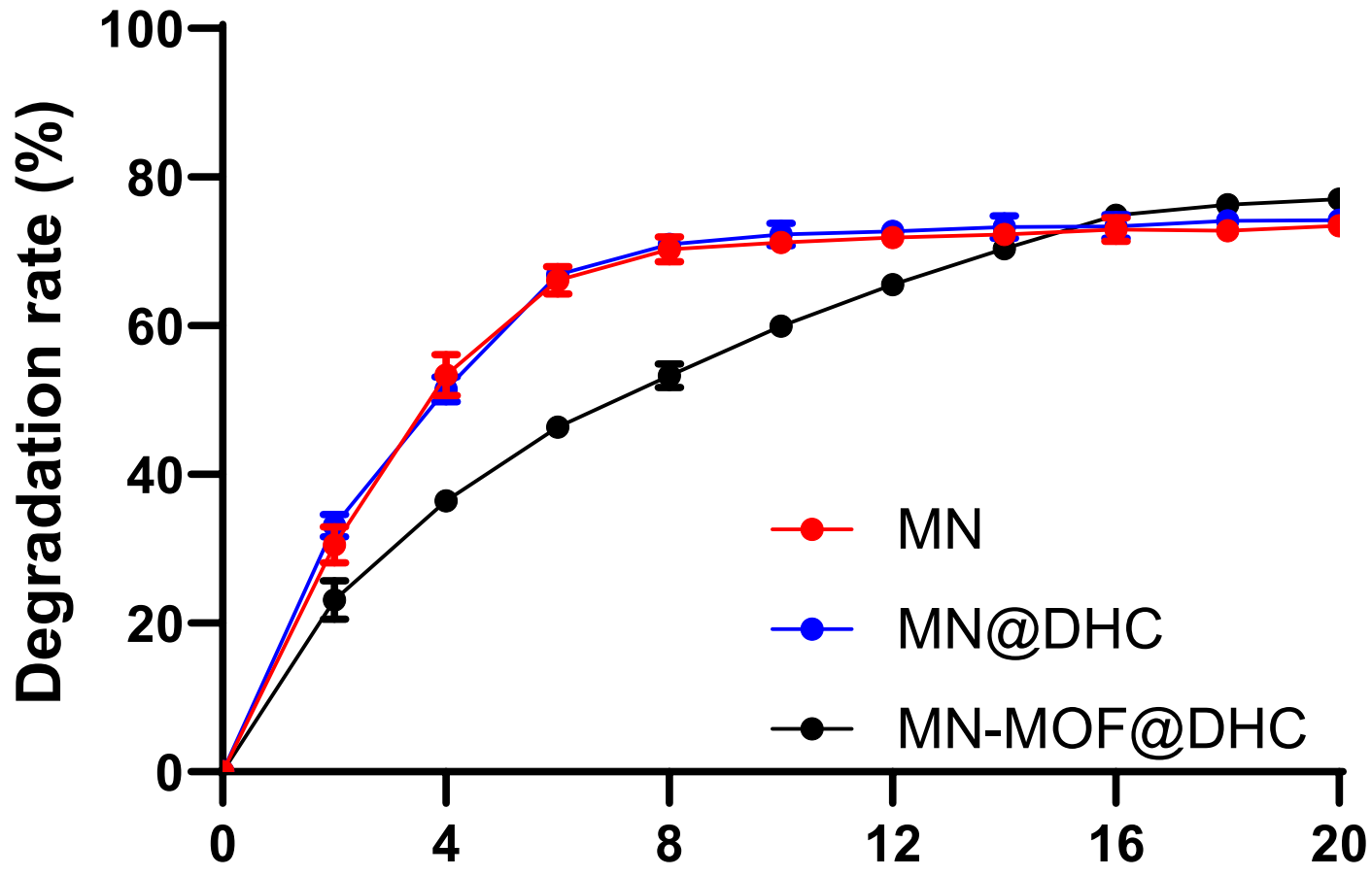

Supplementary Figure 3. The degradation tests of the microneedles in collagenase-2 solution.

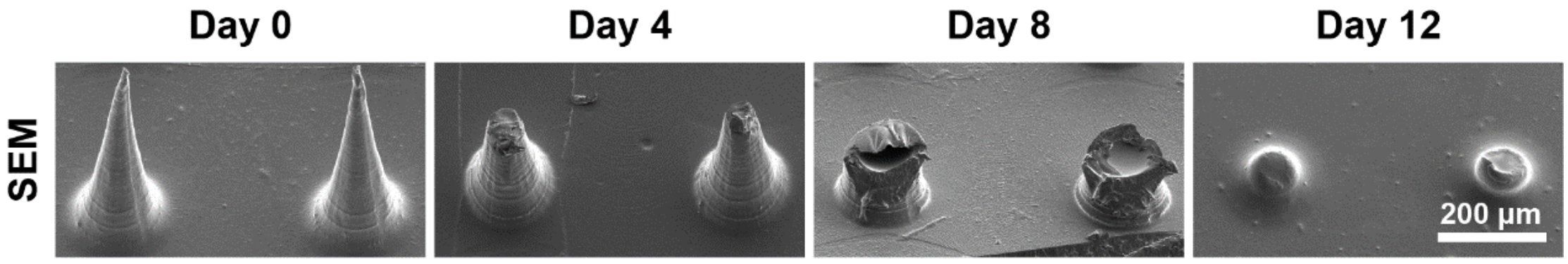

Supplementary Figure 4. The morphological changes of the microneedles by SEM in collagenase-2 solution.

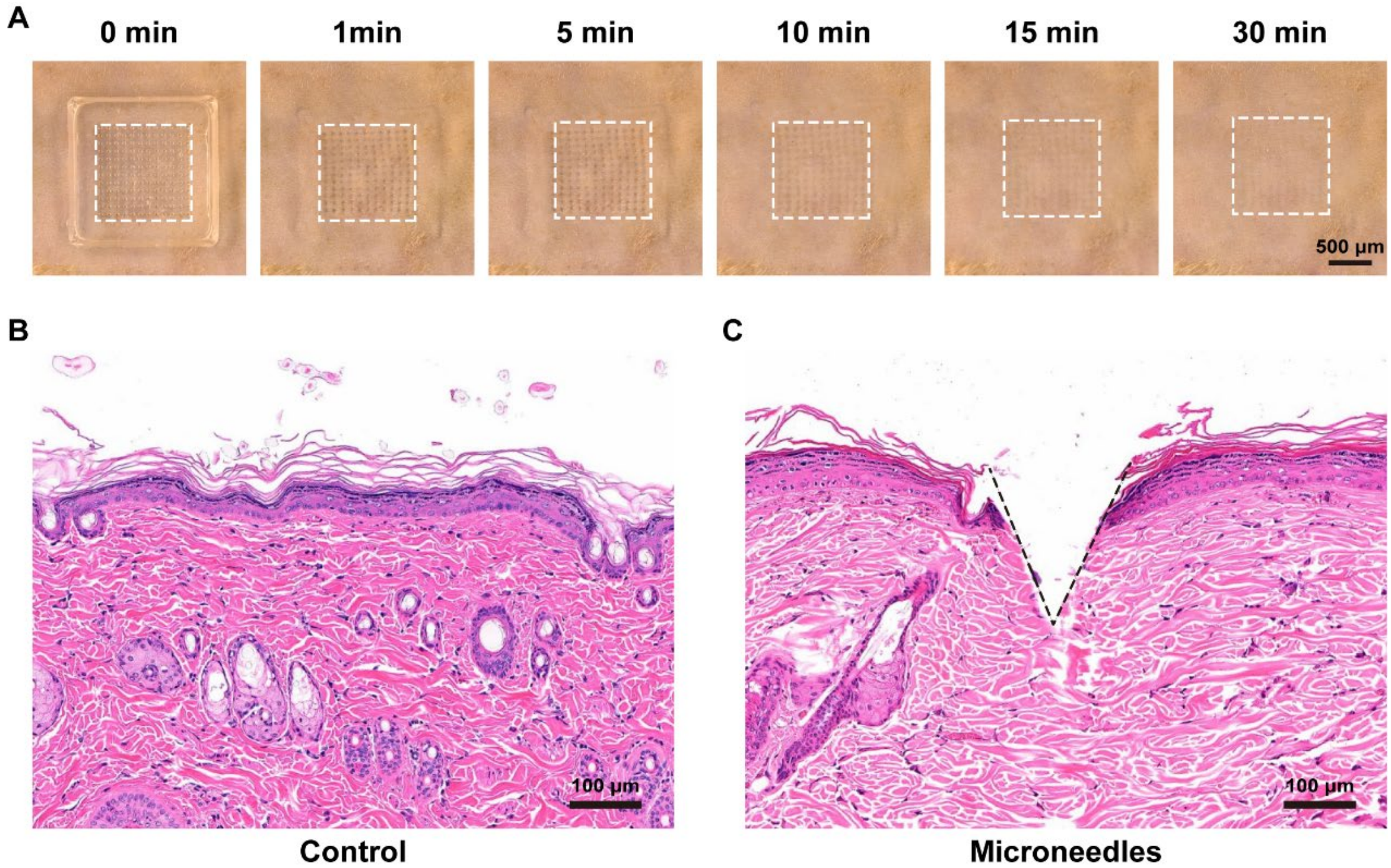

Supplementary Figure 5. Penetration capacity in rat skin of microneedle patches. (A) Photographs of rat skin tissues treated with microneedle patches in different times. (B) Representative image of H&E staining rat skin tissue with or without microneedle patch insertion.

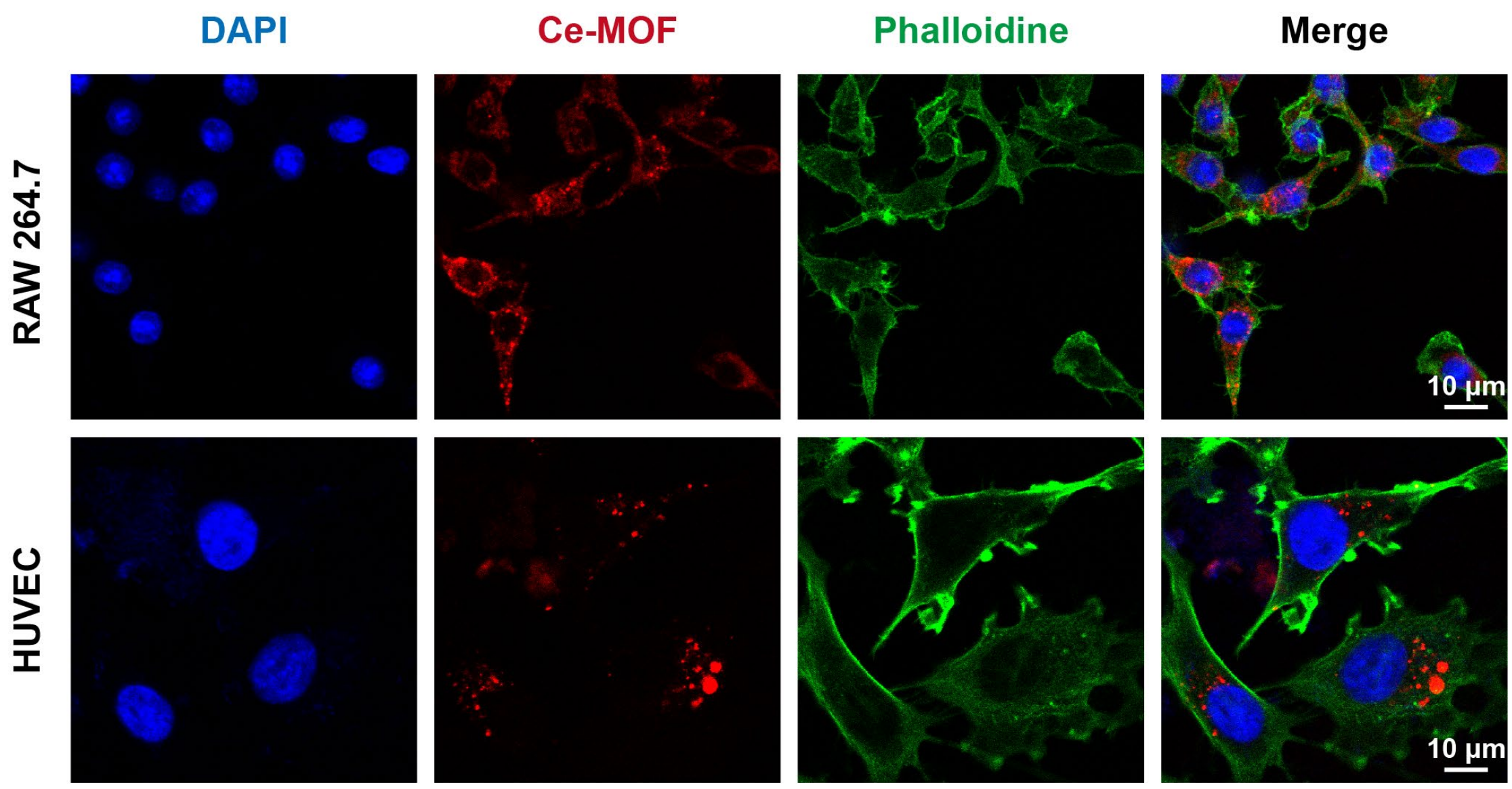

Supplementary Figure 6. Representative images showing the efficiency of cellular uptake of Ce-MOF.

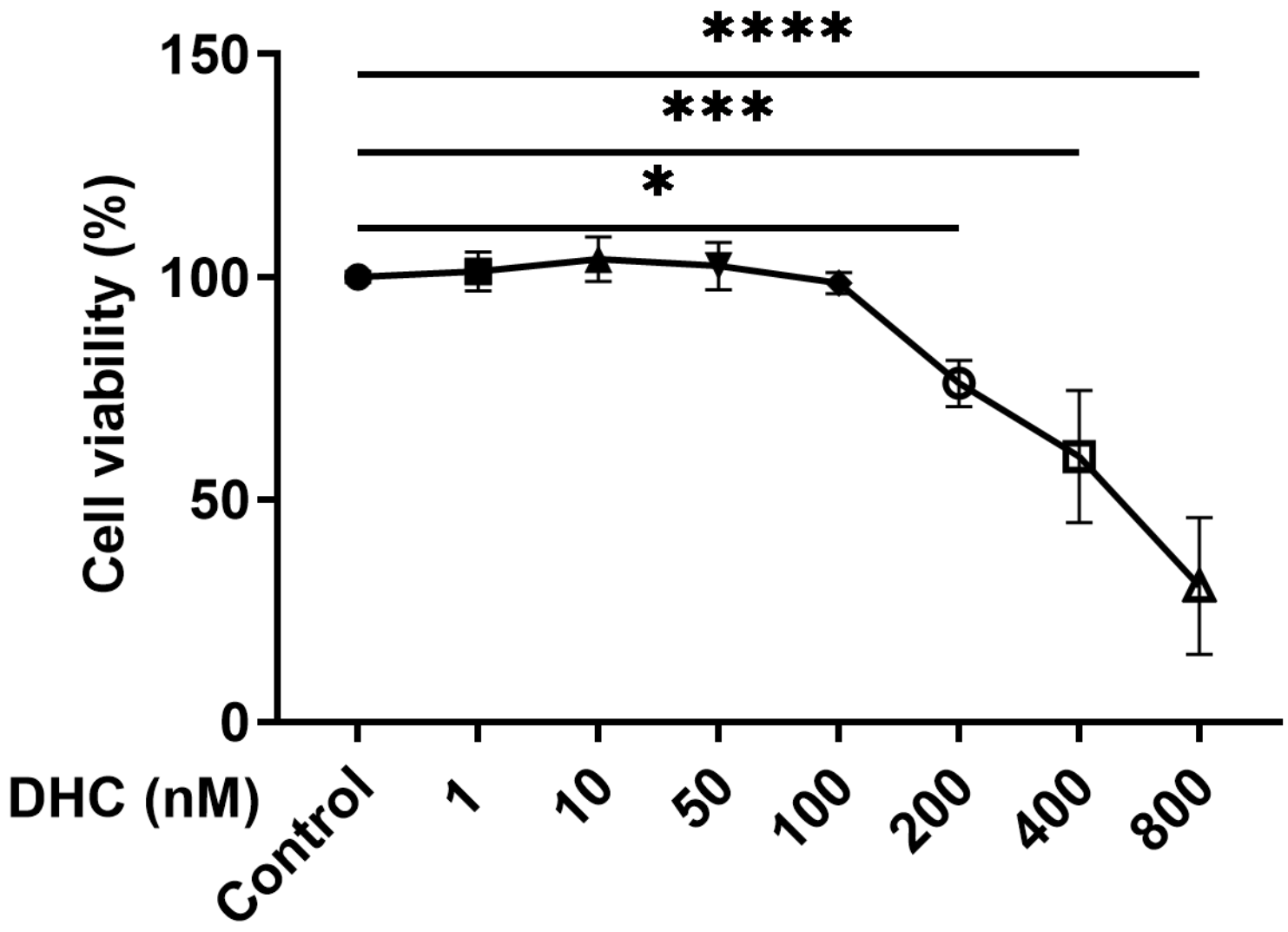

Supplementary Figure 7. The optimal concentration of DHC was determined utilizing the CCK-8 assay. (n = 3, error bars, means ± SD; all analyses were done using one-way ANOVA with Tukey's post hoc test *P < 0.05 , **P < 0.01, ***P < 0.001 and **** P< 0.0001).

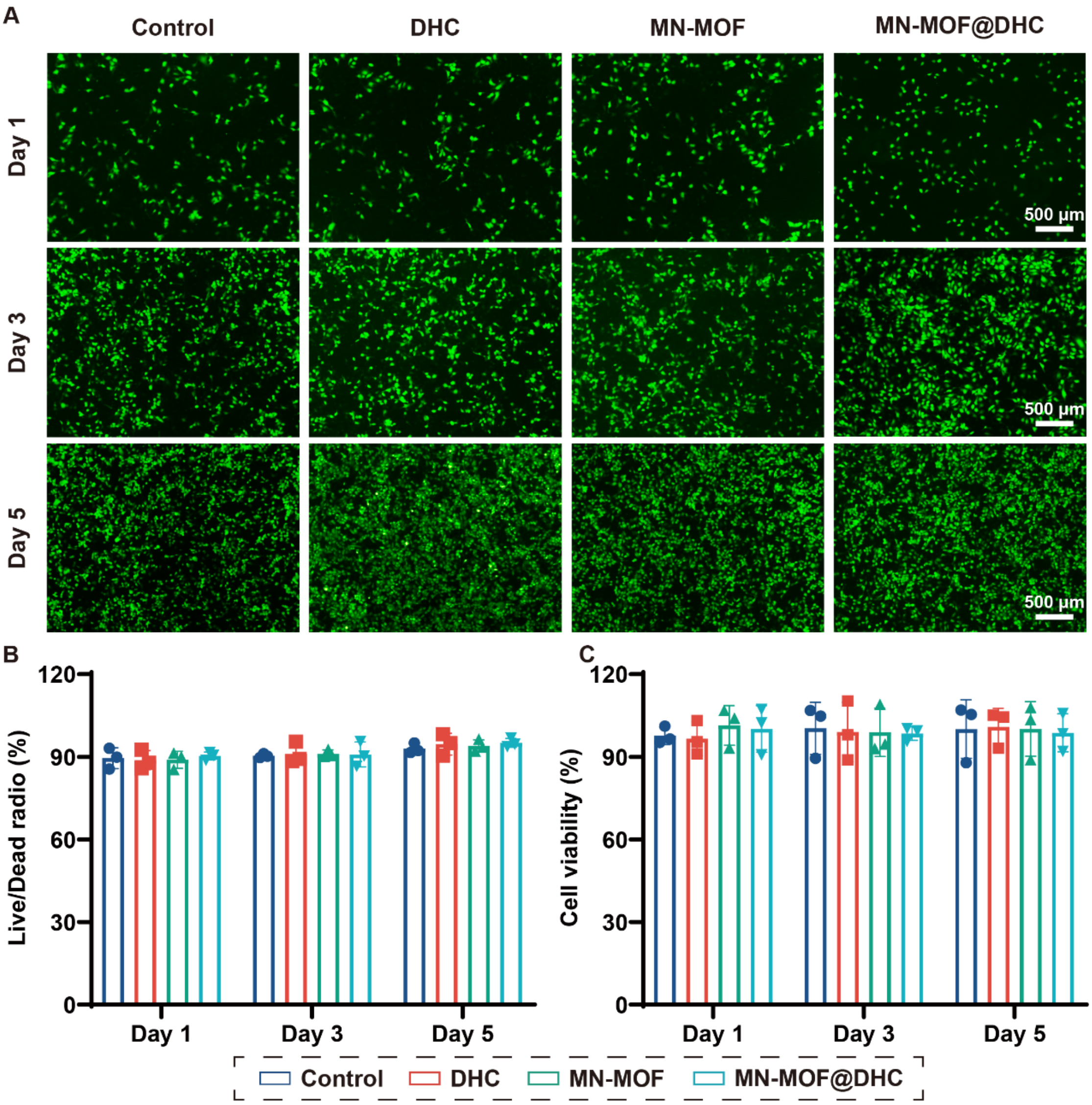

Supplementary Figure 8. Biocompatibility of MN-MOF@DHC. (A) Live/dead staining of HUVECs after treatment with DHC, MN-MOF and MN-MOF@DHC on day 1, 3 and 5. (B) Quantification of Live/dead staining. (C) The viability of HUVECs after treatment with DHC, MN-MOF and MN-MOF@DHC was determined using the CCK-8 assay on day 1, 3, and 5. (n = 3, error bars, means ± SD; all analyses were done using one-way ANOVA with Tukey's post hoc test $^*P < 0.05$, $^{**}P < 0.01$, $^{***}P < 0.001$ and $^{****} P< 0.0001$).

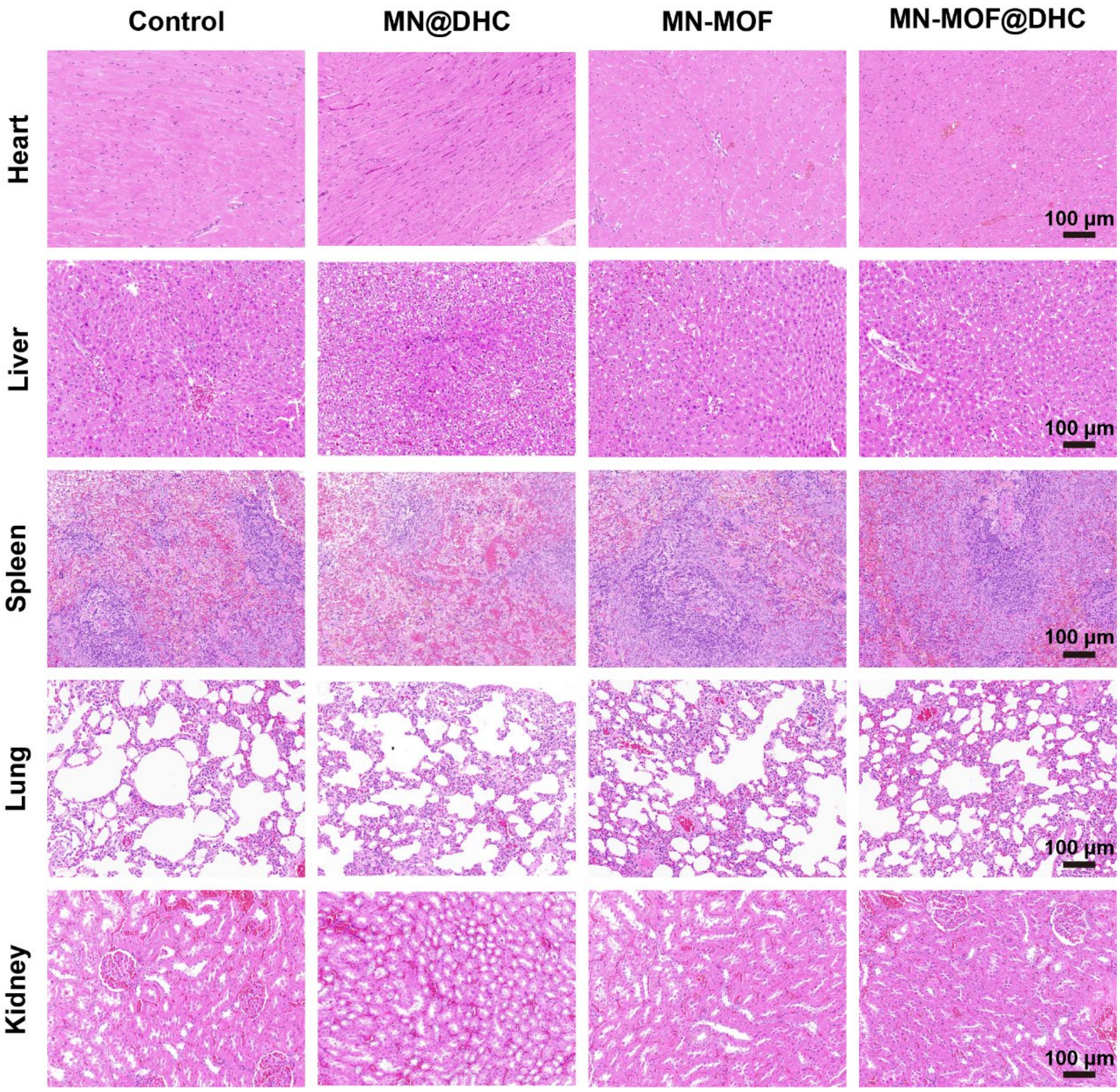

Supplementary Figure 9. Biocompatibility of MN-MOF@DHC in vivo. Representative images of H&E staining in the heart, liver, spleen, lung, and kidney tissues among different groups.

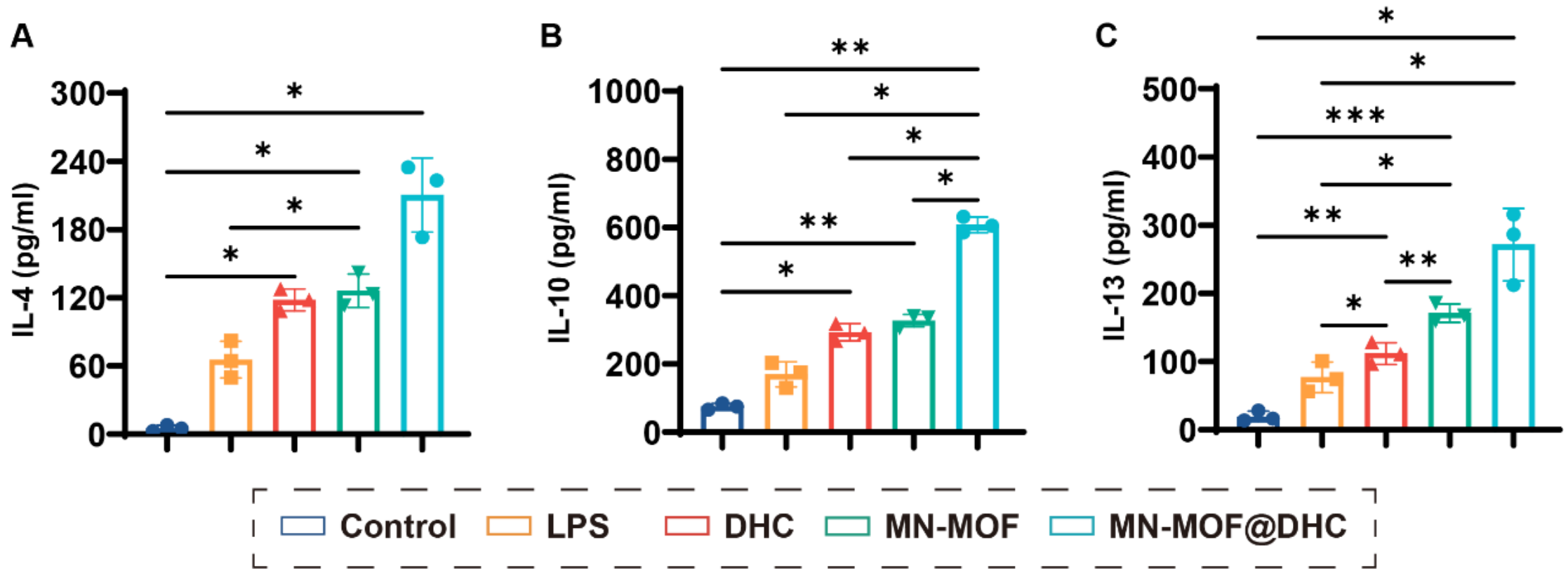

Supplementary Figure 10. Detection of IL-4, IL-10 and IL-13 secreted by macrophages through ELISA. (n = 3, error bars, means ± SD; all analyses were done using one-way ANOVA with Tukey's post hoc test *P < 0.05 , **P < 0.01, ***P < 0.001 and **** P< 0.0001).

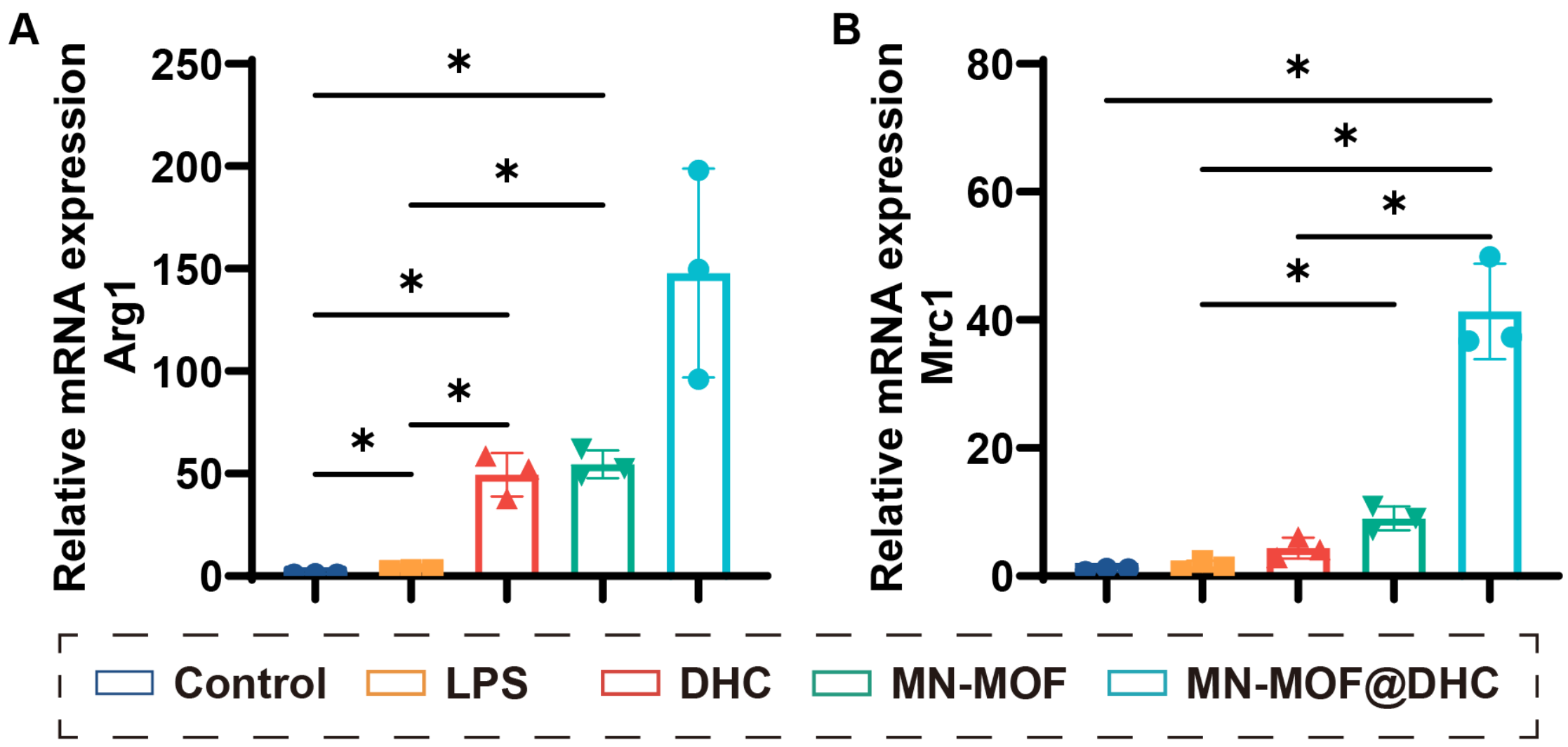

Supplementary Figure 11. RT-PCR was performed to determine the gene expression of Arg1 and Mrc1. (n = 3, error bars, means ± SD; all analyses were done using one-way ANOVA with Tukey's post hoc test *P < 0.05 , **P < 0.01, ***P < 0.001 and **** P< 0.0001).

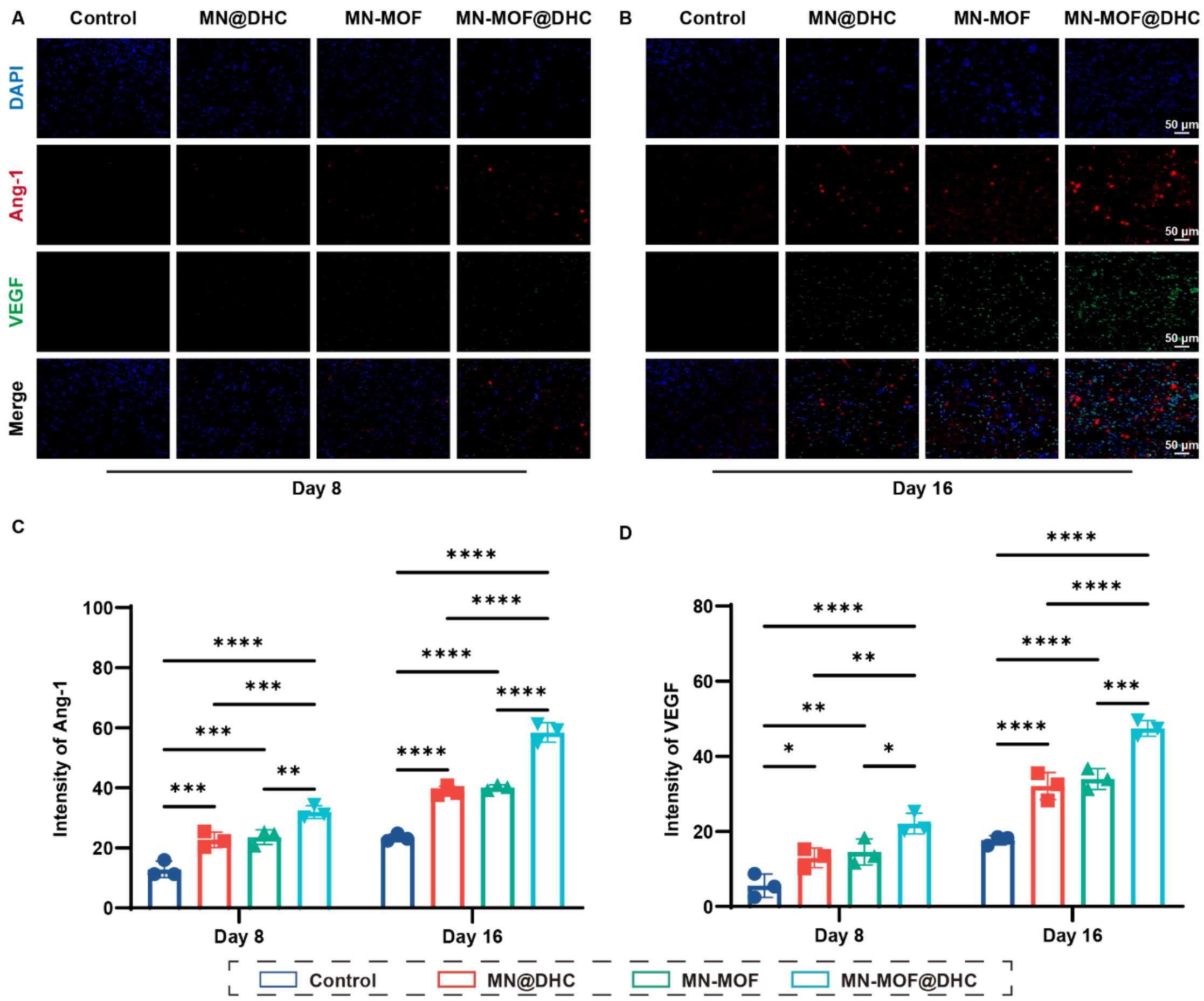

Supplementary Figure 12. In vivo assessment of the angiogenic properties of MN-MOF@DHC. (A-B) Immunofluorescence staining images of Ang-1 and VEGF captured on days 8 and 16. (C-D) Semiquantitative analysis of Ang-1 and VEGF fluorescence intensity on day 8 and 16. (n = 3, error bars, means ± SD; all analyses were done using one-way ANOVA with Tukey's post hoc test *P < 0.05 , **P < 0.01, ***P < 0.001 and **** P< 0.0001).

Supplementary Table 1: Primer sequences of quantitative polymerase chain reaction.

**Gene**	**Sense (5'-3')**	**Antisense (5'-3')**
Arg1	CTCCAAGCCAAAGTCCTTAGAG	GGAGCTGTCATTAGGGACATCA
Mrc1	CTCTGTTCAGCTATTGGACGC	TGGCACTCCCAAACATAATTTGA
GAPDH	ACCCAGAAGACTGTGGATGG	CACATTGGGGGTAGGAACAC
